# Enhancing Green Ammonia Electrosynthesis Through Tuning Sn Vacancies in Sn-Based MXene/MAX Hybrids

**DOI:** 10.1007/s40820-023-01303-2

**Published:** 2024-01-16

**Authors:** Xinyu Dai, Zhen-Yi Du, Ying Sun, Ping Chen, Xiaoguang Duan, Junjun Zhang, Hui Li, Yang Fu, Baohua Jia, Lei Zhang, Wenhui Fang, Jieshan Qiu, Tianyi Ma

**Affiliations:** 1 Key Laboratory for Green Synthesis and Preparative Chemistry of Advanced Materials of Liaoning Province, College of Chemistry, Institute of Clean Energy Chemistry, Liaoning University, Shenyang, 110036 People’s Republic of China; 2 State Key Laboratory of Clean and Efficient Coal Utilization, Taiyuan University of Technology, Taiyuan, 030024 People’s Republic of China; 3 School of Chemistry and Chemical Engineering, Anhui University, Hefei, 230601 People’s Republic of China; 4 School of Chemical Engineering, The University of Adelaide, Adelaide, SA 5005 Australia; 5 State Key Laboratory of High-Efficiency Utilization of Coal and Green Chemical Engineering, College of Chemistry and Chemical Engineering, Ningxia University, Yinchuan, 750021 Ningxia People’s Republic of China; 6 School of Science, RMIT University, Melbourne, VIC 3000 Australia; 7 Guangdong Provincial Key Laboratory of Advanced Energy Storage Materials, School of Chemistry and Chemical Engineering, South China University of Technology, Guangzhou, 510640 People’s Republic of China; 8 College of Chemical Engineering, State Key Laboratory of Chemical Resource Engineering, Beijing University of Chemical Technology, Beijing, 100029 People’s Republic of China

**Keywords:** Green ammonia synthesis, N_2_ electroreduction, Renewable energy, Sn, MXene/MAX hybrid

## Abstract

Renewable energy driven N_2_ electroreduction with air as nitrogen source holds great promise for realizing scalable green ammonia production. However, relevant out-lab research is still in its infancy. Herein, a novel Sn-based MXene/MAX hybrid with abundant Sn vacancies, Sn@Ti_2_CT_*X*_/Ti_2_SnC–V, was synthesized by controlled etching Sn@Ti_2_SnC MAX phase and demonstrated as an efficient electrocatalyst for electrocatalytic N_2_ reduction. Due to the synergistic effect of MXene/MAX heterostructure, the existence of Sn vacancies and the highly dispersed Sn active sites, the obtained Sn@Ti_2_CT_*X*_/Ti_2_SnC–V exhibits an optimal NH_3_ yield of 28.4 µg h^−1^ mg_cat_^−1^ with an excellent FE of 15.57% at − 0.4 V versus reversible hydrogen electrode in 0.1 M Na_2_SO_4_, as well as an ultra-long durability. Noticeably, this catalyst represents a satisfactory NH_3_ yield rate of 10.53 µg h^−1^ mg^−1^ in the home-made simulation device, where commercial electrochemical photovoltaic cell was employed as power source, air and ultrapure water as feed stock. The as-proposed strategy represents great potential toward ammonia production in terms of financial cost according to the systematic technical economic analysis. This work is of significance for large-scale green ammonia production.

**Supplementary Information:**

The online version contains supplementary material available at 10.1007/s40820-023-01303-2.

## Highlights

- Sn-based MAX/MXene hybrids with abundant Sn vacancies, Sn@Ti_2_CT_*X*_/Ti_2_SnC–V, fabricated by controlled etching method, are demonstrated to be an excellent electrocatalyst for N_2_ electroreduction.
- An economic “NH_3_ farm” has been developed based on Sn@Ti_2_CT_*X*_/Ti_2_SnC–V electrode, demonstrated by a commercial electrochemical photovoltaic cell, which may open a novel avenue for solar energy-driven synthesis of ammonia directly from air and water.
- The potential of the “NH_3_ farm” was demonstrated by a systematic technical economic analysis.

**Supplementary Information:**

The online version contains supplementary material available at 10.1007/s40820-023-01303-2.

## Graphical abstract

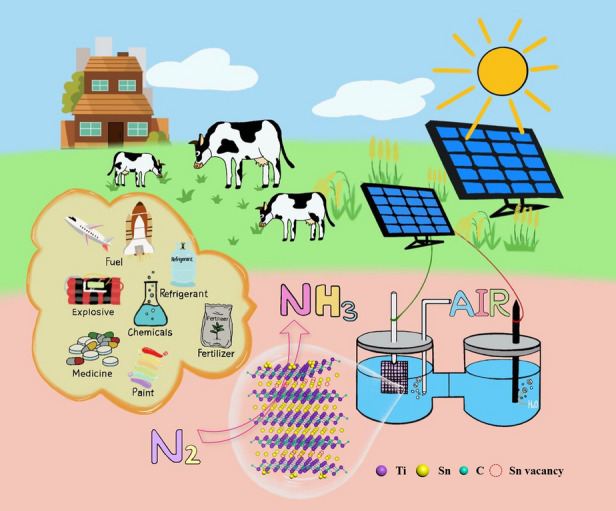

## Introduction

Ammonia (NH_3_), as an important inorganic chemical, is not only widely used in industrial manufacture such as fertilizer, plastics and refrigerants, but also in the field of energy storage and conversion, especially the hydrogen storage [1–3]. Up to now, the industrial NH_3_ production mainly relies on the Haber Bosch (H-B) process, which requires harsh reaction conditions (300–600 °C, 150–350 atm), simultaneously generates enormous energy consumption and CO_2_ emission [4–7]. Considering energy and environmental factors, it is necessary to explore a sustainable and green method for NH_3_ synthesis [8, 9].

Electrocatalytic N_2_ reduction (ENRR) can directly use N_2_ and ultrapure water as raw materials to realize NH_3_ production when powered by electricity, which has become the most promising substitute for the energy-intensive H-B process [10–13]. However, the extremely low Faradic efficiency and NH_3_ yield are still far away from the requirements of practical applications due to the high bond energy of N**≡**N and the drastic competitive hydrogen evolution reaction (HER) [14–17]. The key to solve these problems is to design and fabricate electrocatalysts with high ENRR selectivity and activity but inert HER activity [18–20].

Among various metal-based electrocatalysts that have been widely used in ENRR, Sn-based materials exhibit great potentials for large-scale electrocatalytic NH_3_ production [21–23]. As a P-block carbon group metal, Sn exhibit relatively high ENRR activity and selectivity due to its partially occupied p orbitals, which can provide electrons to the unoccupied anti-bonding orbitals of N_2_ for effectively activating N≡N triple bond [24]. Significantly, the vacant p orbitals endows Sn with inert HER activity [24, 25]. Meanwhile, the advantages of abundant reserves, environmental friendliness and low cost also make Sn suitable for practical use [26]. However, its ENRR performance is seriously impeded by the inferior conductivity, agglomeration and the limited electron transfer rate. In light of this, highly dispersed Sn to substrates with good conductivity is a wisdom strategy to improve its ENRR performance. MXenes, as a kind of two-dimensional transition metal carbide/nitride materials, are generally prepared by selectively etching “A” atomic layers from their parent MAX phases[27], which possess unique properties of being electrocatalysts or substrates, such as tunable components, high specific surface area and outstanding electrical conductivity [28, 29]. When combined with MAX, the heterostructure hybrid will be featured with more abundant active sites, such as defects, vacancies, adjustable electronic structures/electrical conductivity, improved structural and chemical stability, which is favor for achieving high NH_3_ yield and FE [4, 30, 31].

Inspired by the “Hydrogen Farm Project” strategy proposed by Li and coworkers, which opens an economical way for practical hydrogen production and significantly improved the utilization of renewable energy [32], we turn our attention from laboratory research to build a small-scale “NH_3_ farm”. The ENRR possesses the merits of simple device, easy operation and low investment, thus suitable for building an economical “NH_3_ farm” with adjustable scale when powered by solar panels [33]. This strategy would be more promising if air could be used instead of high-purity N_2_, which further reducing the energy consumption and CO_2_ emission. However, the presence of O_2_ in the air puts higher demands on the stability and selectivity of the catalysts for achieving high ENRR performance.

Herein, we for the first time synthesized a MXene/MAX hybrid with highly dispersed Sn and Sn vacancies, denoted as Sn@Ti_2_CT_*X*_/Ti_2_SnC–V, by a simply controlled HF etching method. This hybrid demonstrates a high NH_3_ yield rate of 28.4 µg h^−1^ mg_cat_^−1^ at − 0.4 V versus RHE with an FE of 15.57% in neutral electrolyte with the help of the synergistic effect of fully exposed Sn active centers, Sn vacancies, and MXene/MAX heterostructure. Then a demonstrator for out-lab green NH_3_ production was constructed, of which a commercial photovoltaic panel was used for directly converting solar energy to electricity to drive the ENRR process, and pre-purified air was employed as N_2_ source. Notably, the obtained maximum NH_3_ production rate of 10.53 µg h^−1^ mg^−1^ obtained by the present demonstrator does imply its promising potential in establishing “NH_3_ farm” for the next generation energy conversion and storage. The economic feasibility of the present catalytic system was further proved by technical economic analysis.

## Experimental

### Preparation of Sn@Ti_2_SnC MAX

Sn@Ti_2_SnC was successfully prepared by a pressure-free method [34, 35]. Typically, 9.57 g Ti powder (99.8%, 300 mesh), 24.0 g Sn powder (99.5%, 200 mesh) and 2.40 g graphite powder (99.95%, 500 mesh) were mixed thoroughly by ball-milling technique. Then, the mixture was placed into graphite molds pre-sprayed with BN layer and sintered in Ar atmosphere at 1200 °C for 2 h. Finally, the product was washed with ultrapure water for three times and vacuum dried at 60 °C to obtain Sn@Ti_2_SnC MAX.

### Preparation of Sn@Ti_2_CT_***X***_/Ti_2_SnC–V

Sn@Ti_2_CT_*X*_/Ti_2_SnC–V was prepared by the traditional HF etching method [36, 37]. Typically, 0.5 g Sn@Ti_2_SnC MAX was dispersed in 120 mL 40% HF solution and etched for a certain time at 25 °C, during which the etching time varies from 1 to 3 h to adjust the morphology and components of the target product. The mixture was centrifuged and washed with ultrapure water for three times to remove residual HF, dried in a vacuum drying oven at 60 °C for 12 h to obtain the Sn@Ti_2_CT_*X*_/Ti_2_SnC–V.

### Preparation of the Working Electrode

The prepared catalyst was dispersed in a mixture of 20 μL Nafion solution (5 wt%), 480 μL ethanol and 500 μL ultrapure water for 1 h to form a uniform ink. 20 µL of catalyst ink droplets were then placed on a 1 × 1 cm^2^ carbon cloth (CC) and vacuum dried for subsequent measurements.

## Results and Discussion

### Characterization

**Fig. 1:**
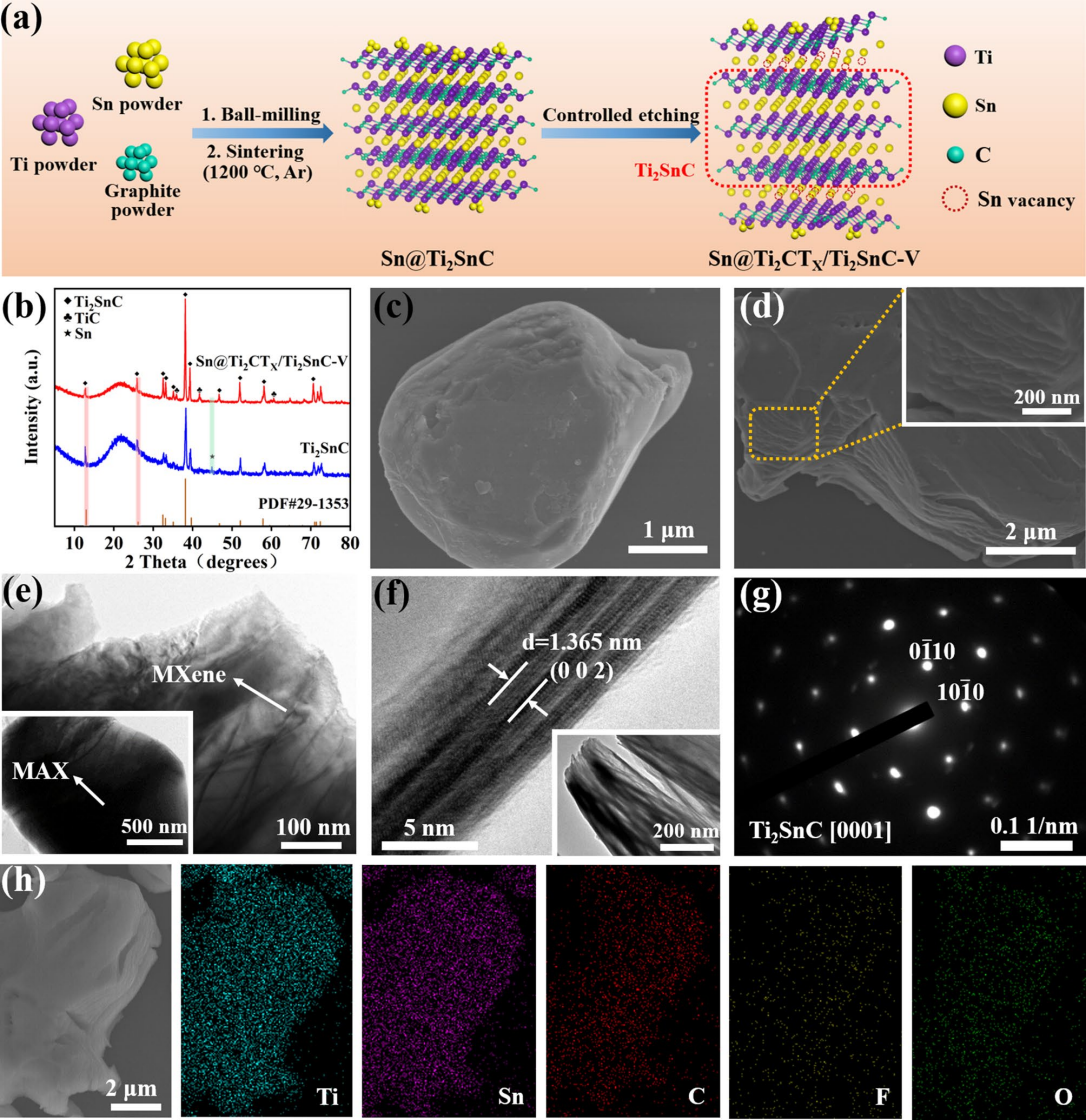
**a** Schematic diagram for the synthesis of Sn@Ti_2_CT_*X*_/Ti_2_SnC–V. **b** XRD patterns of Sn@Ti_2_SnC and Sn@Ti_2_CT_*X*_/Ti_2_SnC–V. **c** SEM image of Sn@Ti_2_SnC MAX. **d** SEM, **e** TEM, **f** HRTEM, **g** SAED pattern, **h** SEM and corresponding element (Ti, Sn, C, F and O) mapping images of Sn@Ti_2_CT_*X*_/Ti_2_SnC–V

Figure 1a illustrates the preparation process of the as-prepared Sn@Ti_2_CT_*X*_/Ti_2_SnC–V electrocatalyst. As shown in Fig. 1a, Sn@Ti_2_SnC MAX was synthesized through the pressure-free method by using Ti, Sn and graphite powder as raw materials. The structure of Ti_2_SnC is considered as Sn atomic layer inserted periodically between Ti_6_C octahedral layers, that is, in Ti_2_SnC, the adjacent Ti–C–Ti bond chain is connected by an Sn atom, forming a layered structure [38]. The traditional HF etching method was employed to synthesize the Sn@Ti_2_CT_*X*_/Ti_2_SnC–V. During the etching process, partial Sn layers are removed from their parent phases Sn@Ti_2_SnC by controlling the etching time to form Sn@Ti_2_CT_*X*_/Ti_2_SnC–V, because the Ti–C covalent bonds in the Ti_2_SnC MAX are stronger than the Sn–Ti bonds [39]. At the same time, part of Sn atoms are etched away to form vacancies, which can act as the active sites of ENRR.

The crystalline phase of Sn@Ti_2_SnC and Sn@Ti_2_CT_*X*_/Ti_2_SnC–V was investigated by the X-ray diffraction (XRD). As it is shown in Fig. 1b, the XRD pattern of the as-synthesized Sn@Ti_2_SnC sample shows characteristic peaks at 2*θ* of 12.72°, 25.89°, 32.63°, 33.27°, 35.03°, 38.28°, 39.45°, 46.95°, 52.13°, 58.29° and 70.80° corresponding to the (002), (004), (100), (101), (102), (103), (006), (105), (106), (110) and (109) crystal planes of Ti_2_SnC (JCPDS 29-1353), indicating the successful synthesis of Ti_2_SnC MAX phase [40, 41]. The diffraction peak located at 44.9° originates from (211) plane of Sn metal (JCPDS 04-0673) [42]. Besides, no typical peaks stem from TiC and the Ti-Sn compounds are found, indicating the present synthesis method can avoid the formation of by-products. After being etched with HF solution, the presence of Ti_2_CT_*X*_ is verified by the emergence of three typical peaks at 36.80°, 41.91° and 60.75°. Notably, the intensity of the peaks at 2*θ* of 38.28° and 39.45° is sharply increased, which demonstrates a better crystal structure after etching. The disappearance of the characteristic peak at 2*θ* of 44.9° indicates that part of Sn metal are etched away or their particle size are too small to be detected after etching with HF [40]. Compared with those of Sn@Ti_2_SnC, the (002) and (004) peaks of Sn@Ti_2_CT_*X*_/Ti_2_SnC–V exhibit slight shift toward small angle, indicating the layer spacing becomes larger after etching. This increased interlayer spacing is more conducive to the electrolyte infiltration during the whole ENRR process.

The morphology and microstructure of the as-prepared Sn@Ti_2_SnC and Sn@Ti_2_CT_*X*_/Ti_2_SnC–V were examined by scanning electron microscopy (SEM) and transmission electron microscopy (TEM). As shown in Fig. 1c, the original Sn@Ti_2_SnC MAX shows a plate-like structure [43]. In addition, according to the mapping and energy-dispersive spectrometer (EDS) results of Sn@Ti_2_SnC (Fig. S1 and Table S1), the atomic ratio of Ti: Sn is 19.02: 10.03, which may attribute to the excess Sn decorated on the surface of the as-synthesized Ti_2_SnC. These Sn can act as active sites for enhancing the ENRR performance. Figure 1d illustrates the SEM image of the obtained Sn@Ti_2_CT_*X*_/Ti_2_SnC–V, which shows a two-dimensional (2D) layered structure with a larger layer spacing than that of Sn@Ti_2_SnC, attributing to the partially removing of the Sn layer by HF etching. TEM images of Sn@Ti_2_CT_*X*_/Ti_2_SnC–V further revealed that abundant cracks existed in 2D layered structures, as shown in Fig. 1e, which helps to expose more active sites for a better electrocatalytic activity. From the HRTEM image of Sn@Ti_2_CT_*X*_/Ti_2_SnC–V (Fig. 1f), the 1.365 nm crystal plane spacing is ascribed to the (002) plane of Sn@Ti_2_CT_*X*_/Ti_2_SnC–V, which has been broadened after HF etching [44]. Figure 1g depicts the typical selection region electron diffraction (SAED) pattern of Sn@Ti_2_CT_*X*_/Ti_2_SnC–V, which is indexed as (0001) plane of Ti_2_SnC [45]. In addition, the results of EDS (Fig. 1h) verifies that the F and O terminations were uniformly dispersed throughout the Ti_2_CT_*X*_/Ti_2_SnC surface.

**Fig. 2:**
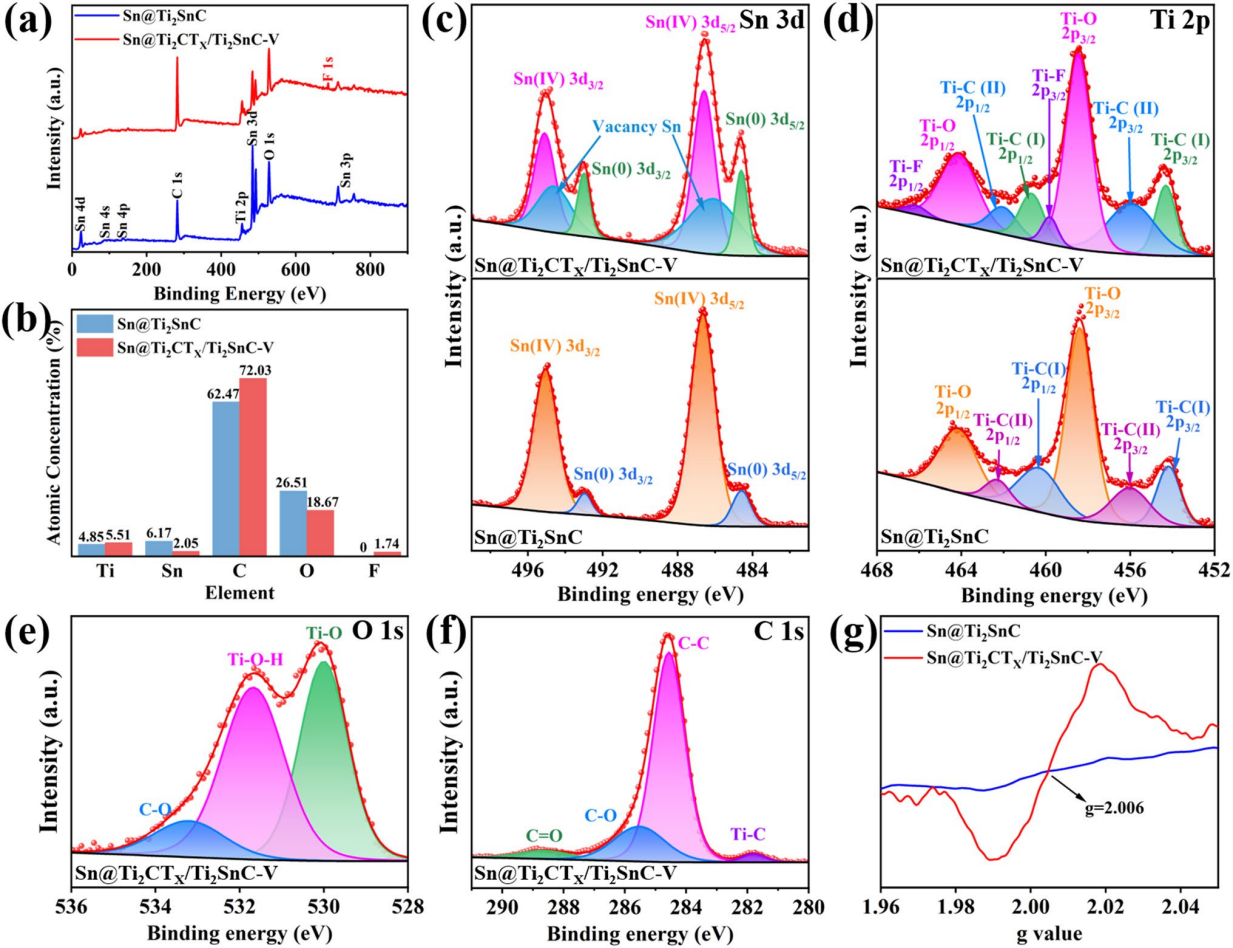
Surface chemical environments of Sn@Ti_2_CT_*X*_/Ti_2_SnC–V and Sn@Ti_2_SnC. **a** Survey scan spectra. **b** Atomic concentration. **c** High-resolution XPS spectra of Sn 3*d*, **d** Ti 2*p*, **e** O 1*s* and **f** C 1*s*. **g** EPR spectra

The surface chemical states of the two as-prepared samples were detected by X-ray photoelectron spectroscopy (XPS). As shown in Fig. 2a, the survey scan spectrum of Sn@Ti_2_CT_*X*_/Ti_2_SnC–V shows Ti 2*p*, Sn 3*d*, C 1*s*, F 1*s* and O 1*s* peaks, which are consistent with the results of the EDS (Fig. 1h). The corresponding element quantification for Sn@Ti_2_SnC and Sn@Ti_2_CT_*X*_/Ti_2_SnC–V are shown in Fig. 2b. As it is demonstrated that after etching with HF solution, the content of Sn drops from 6.17 to 2.05 at% as some of Sn atoms are removed from the Sn@Ti_2_SnC. The high-resolution Sn 3*d* XPS spectrum of Sn@Ti_2_SnC (Fig. 2c, lower) displays four peaks at 495.0, 492.9, 486.6 and 484.5 eV, arising from the Sn(IV) 3*d*_3/2_, Sn(0) 3*d*_3/2_, Sn(IV) 3*d*_5/2_ and Sn(0) 3*d*_5/2_, respectively. Meanwhile, as shown in the Sn 3*d* spectrum of Sn@Ti_2_CT_*X*_/Ti_2_SnC–V (Fig. 2c, upper), the characteristic peaks of Sn(IV) 3*d*_3/2_, Sn(0) 3*d*_3/2_, Sn(IV) 3*d*_5/2_ and Sn(0) 3*d*_5/2_ are well presented at 495.0, 493, 486.6 and 484.6 eV. These typical Sn 3*d* peaks of Sn@Ti_2_CT_*X*_/Ti_2_SnC–V exhibit ca. 0.1 eV shift toward high binding energy comparing with those of Sn@Ti_2_SnC, indicating that the etching process generates the decrease of the electron density of the 3*d* orbital in Sn atoms, which is conducive to boosting the adsorption of N_2_ [6]. Besides, the characteristic peaks at 493.6 and 485.2 eV in the XPS spectrum of Sn@Ti_2_SnC/Ti_2_CT_*X*_-V are attributed to the Sn vacancy [46, 47]. Defect engineering is a feasible strategy to improve the catalytic performance of ENRR by effectively adjusting the electronic and surface properties of catalysts [48–51]. The A-layer metal Sn of MAX is etched during the etching process, thus forming Sn vacancies. The introduction of Sn vacancies can significantly tune the electronic structure of the catalyst and adjust the surface adsorption of the reaction intermediates, thus further improving the electrocatalytic activity [52, 53]. Notably, these Sn vacancies are the active sites for chemisorption of N_2_, due to the accumulation of a large number of local electrons around them [6]. At the same time, the empty p orbitals in Sn without occupied electrons are conducive to inhibiting HER. As shown in the high-resolution Ti 2*p* XPS spectra of Sn@Ti_2_CT_*X*_/Ti_2_SnC–V (Fig. 2d, upper), the fitting peaks located at 455.9 and 462.1 eV are assigned to the Ti 2*p* peaks of Ti–C(II), while the peaks at 454.2/460.7 eV are corresponding to Ti–C(I) bond [54]. The Ti 2*p* peaks at bonding energy of 464.1 and 458.5 eV can be attributed to the Ti–O bond in the surface of Sn@Ti_2_CT_*X*_/Ti_2_SnC–V, which formed during the washing process [55]. The peaks of Ti–C bond in the XPS spectra of Sn@Ti_2_CT_*X*_/Ti_2_SnC–V are negative shifted by 0.1 eV compared with those in the spectra of Sn@Ti_2_SnC (Fig. 2d, lower), demonstrating the increase IN electron density of Ti 2*p* obtial. The high-resolution O 1*s* XPS spectrum of Sn@Ti_2_CT_*X*_/Ti_2_SnC–V (Fig. 2e) displays three peaks at 529.96, 531.66 and 533.16 eV, arising from the Ti–O, Ti–O–H and C–O bonds, respectively [56]. According to the reported experimental and theoretical studies, MXenes with O terminals exhibit superior stability due to the higher oxidation state of Ti bonding with O than F terminal [57]. The high-resolution C 1*s* spectrum of Sn@Ti_2_CT_*X*_/Ti_2_SnC–V can be divided into four peaks at 281.76, 284.56, 285.56, and 288.66 eV, corresponding to Ti–C, C–C, C–O, and C=O, as depicted in Fig. 2f [58]. In addition, the existence of Sn vacancies was further confirmed by electron paramagnetic resonance (EPR) spectroscopy analysis (Fig. 2g). According to the previous report [59, 60], the formation of Ti vacancies would present a paramagnetic center at *g* = 2.004–2.005. Nevertheless, this signature of Ti vacancies has not been observed in our samples. According to XPS and EPR analysis, the partially etched Sn@Ti_2_SnC with Sn vacancies were successfully prepared. Sn vacancies optimize the electronic structure of the Sn@Ti_2_CT_*X*_/Ti_2_SnC–V, thereby enhancing the charge transfer and reducing the adsorption energetics of the electrocatalytic reaction intermediates, thus improving the ENRR performance [61].

### ENRR Performance

**Fig. 3:**
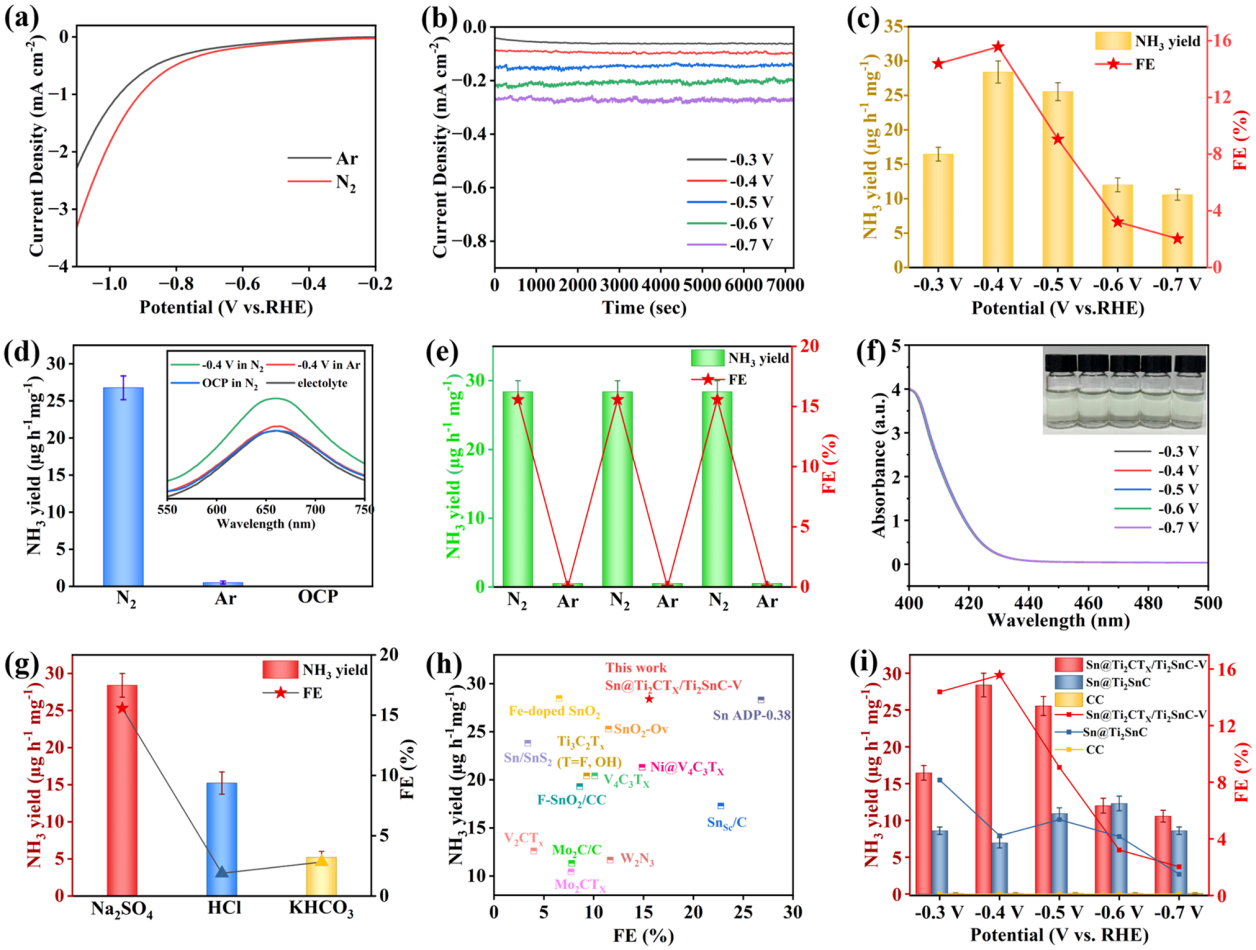
**a** LSV curves of Sn@Ti_2_CT_*X*_/Ti_2_SnC–VC–V in Ar- and N_2_- saturated 0.1 M Na_2_SO_4_. **b** CA results of Sn@Ti_2_CT_*X*_/Ti_2_SnC–V obtained in N_2_- saturated 0.1 M Na_2_SO_4_ at different potentials. **c** NH_3_ yields and FEs at selected potentials. **d** NH_3_ yields and corresponding UV–Vis absorption spectra (inset) of electrolytes after electrolysis for 2 h under different control conditions. **e** NH_3_ yields and FEs of Sn@Ti_2_CT_*X*_/Ti_2_SnC–V obtained in the electrolyte saturated with N_2_ and Ar in alternating 2 h cycles at -0.4 V (vs. RHE), respectively. **f** UV–Vis absorption spectra of the electrolytes after 2 h ENRR testing at selected potentials determined by the Watt and Chrisp method. The inset shows the chromogenic reaction of the indicator with N_2_H_4_·H_2_O. **g** NH_3_ yields and FEs of Sn@Ti_2_CT_*X*_/Ti_2_SnC–V at − 0.4 V (vs. RHE) in various electrolytes. **h** NH_3_ yield and FE diagram of different Sn- and MXene-based ENRR catalysts [1–12]. **i** NH_3_ yields and FEs of Sn@Ti_2_CT_*X*_/Ti_2_SnC–V, Sn@Ti_2_SnC and CC in N_2_- saturated 0.1 M Na_2_SO_4_ electrolyte at corresponding potentials

The ENRR performance of Sn@Ti_2_CT_*X*_/Ti_2_SnC–V sample were investigated in a gas-tight two-compartment H-cell with ultra-high purity N_2_ saturated electrolyte. As shown in Fig. 3a, the linear sweep voltammetry (LSV) curves of the catalyst in 0.1 M Na_2_SO_4_ electrolyte saturated with Ar and N_2_ exhibit similar shape. However, a higher current density is achieved in the N_2_-saturated electrolyte when the potential is more negative than − 0.2 V versus reversible hydrogen electrode (vs. RHE), implying that Sn@Ti_2_CT_*X*_/Ti_2_SnC–V/CC possesses catalytic activity for ENRR. Then, the ENRR performance of Sn@Ti_2_CT_*X*_/Ti_2_SnC–V was examined by Chronoamperometric (CA) measurements at different potentials for each 2 h (Fig. 3b). Indophenol blue method was used to quantify the concentration of NH_3_ in each electrolyte, corresponding calibration curves are shown in Figs. S2 and S3. The concentration of NH_3_ in the electrolyte was determined by UV–Vis absorption spectra (Fig. S4), thus calculating the average yield of NH_3_ and the corresponding FE. As shown in Fig. 3c, an increase and subsequent decline in activity and selectivity for ENRR was observed as the potential became more negative. The Sn@Ti_2_CT_*X*_/Ti_2_SnC–V/CC demonstrates an optimal NH_3_ yield of 28.4 µg h^−1^ mg^−1^ with an FE of 15.57% at − 0.4 V (vs. RHE). When potentials are more negative than − 0.4 V (vs. RHE), a notable decrease in NH_3_ yield and FE is observed, suggesting that HER occupies the active sites and becomes dominant. For verifying the reliability of colorimetric method, the concentration of NH_3_ was also determined by ammonia sensitive selective electrode method (Figs. S5 and S6). As depicted in Figs. 3c and S7, within the allowable error range, the yields of NH_3_ measured by this method is basically consistent with those determined by indophenol blue method, indicating that both methods are reliable for the quantitative analysis of NH_3_ in the electrolytes.

In addition, UV–Vis absorption spectra (the inset of Fig. 3d) and corresponding NH_3_ yields (Fig. 3d) manifest no NH_3_ is detected when electrocatalysis is performed over Sn@Ti_2_CT_*X*_/Ti_2_SnC–V in Ar-saturated electrolyte at 0.4 V (vs. RHE) or in N_2_-saturated electrolyte at open-circuit potential (OCP), demonstrating the detected NH_3_ is neither from the electrolyte, nor from the impurities of N_2_ gas, but from the electrocatalysis of N_2_ over Sn@Ti_2_CT_*X*_/Ti_2_SnC–V. A 12-h alternate test in $\text N _{2^-}$ and Ar-saturated 0.1 M Na_2_SO_4_ at − 0.4 V (vs. RHE) was conducted with a 2-h interval (Fig. 3e) to further investigate the N source of the generated NH_3_, which is consistent with the above results. Hydrazine (N_2_H_4_) is an important by-product of NRR process generated by the desorption of the *NH_2_NH_2_ intermediates from the active sites without further hydrogenate, so it is essential to detect the concentration of N_2_H_4_ in the electrolytes to evaluate the selectivity of catalyst [62]. As shown in Figs. 3f and S8, negligible N_2_H_4_ is detected at potentials from − 0.3 to − 0.7 V (vs. RHE), indicating the excellent selectivity of Sn@Ti_2_CT_*X*_/Ti_2_SnC–V for the conversion of N_2_ to NH_3_.

The ENRR performance of Sn@Ti_2_CT_*X*_/Ti_2_SnC–V in both acidic and alkaline electrolytes was studied under the same experimental conditions as that of in Na_2_SO_4_ electrolyte. As shown in Figs. 3g and S9–S12, Sn@Ti_2_CT_*X*_/Ti_2_SnC–V achieves an NH_3_ yield of 15.22 µg h^−1^ mg^−1^ and maximum FE of 1.89% at − 0.4 V (vs. RHE) in 0.1 M HCl solution. This ultra-low FE is mainly due to the adequate proton source in acidic media, which promotes the HER side reaction, simultaneously comprise the selectivity toward ENRR to a large extent. In 0.1 M KHCO_3_ electrolyte, Sn@Ti_2_CT_*X*_/Ti_2_SnC–V achieves an ordinary NH_3_ yield of 5.22 µg h^−1^ mg^−1^ at − 0.4 V (vs. RHE) on account of the lack of proton sources in the alkaline medium, which seriously hinders the hydrogenation process of adsorbed N_2_ on active sites.

Up to now, Sn- and MXene- based materials are intensively studied on ENRR as shown in Fig. 3h and Table S9. Among these catalysts, Sn@Ti_2_CT_*X*_/Ti_2_SnC–V shows satisfactory performance on NH_3_ yield and FE at lower potentials in the neutral electrolyte. For example, the FE of Sn@Ti_2_CT_*X*_/Ti_2_SnC–V is approximately 4.24 times of Sn dendrites (− 0.6 V) [63], 3.89 times of V_2_CT_*x*_ (− 0.7 V) [64] and 1.35 times of SnO_2_–O_v_ (− 0.6 V) [65]. At the same time, the NH_3_ yield of Sn@Ti_2_CT_*X*_/Ti_2_SnC–V is higher than those of most MXenes, which is 1.4 times of Ti_3_C_2_T_*X*_ (20.4 µg h^−1^ mg_cat_^−1^) [66], 2.25 times of V_2_CT_*X*_ (12.6 µg h^−1^ mg_cat_^−1^) [64], and 2.72 times of Mo_2_CT_*X*_ (10.43 µg h^−1^ mg_cat_^−1^) [67]. According to the above comparison, Sn@Ti_2_CT_*X*_/Ti_2_SnC–V is expected to be an excellent substrate and a composite catalyst for application in ENRR.

The ENRR performances of bare carbon cloth (CC), Sn@Ti_2_SnC/CC and Sn@Ti_2_CT_*X*_/Ti_2_SnC–V/CC were tested under the same conditions to evaluate the ENRR activity of different components and the ENRR mechanism of Sn@Ti_2_CT_*X*_/Ti_2_SnC–V. As demonstrated in Fig. 3i, bare CC exhibits no ENRR activity at potentials arranging from − 0.3 to − 0.7 V (vs. RHE). The Sn@Ti_2_SnC MAX phase obtains its highest ammonia yield of 12.3 µg h^−1^ mg^−1^ at − 0.6 V (vs. RHE) which is only 43.9% of that of Sn@Ti_2_CT_*X*_/Ti_2_SnC–V (28.4 µg h^−1^ mg_cat_^−1^) at the applied potential of − 0.4 V (vs. RHE). While its optimal FE (5.36%) at − 0.5 V (vs. RHE) is 34.4% of that obtained by Sn@Ti_2_CT_*X*_/Ti_2_SnC–V at − 0.4 V. Notably, Sn@Ti_2_CT_*X*_/Ti_2_SnC–V achieves an excellent NH_3_ yield at low over-potential of − 0.4 V (vs. RHE), which is almost 4 times of Sn@Ti_2_SnC (6.97 µg h^−1^ mg^−1^), highlighting the important role of Sn vacancies and Ti_2_CT_*X*_ MXene for the improved ENRR activity.

**Fig. 4:**
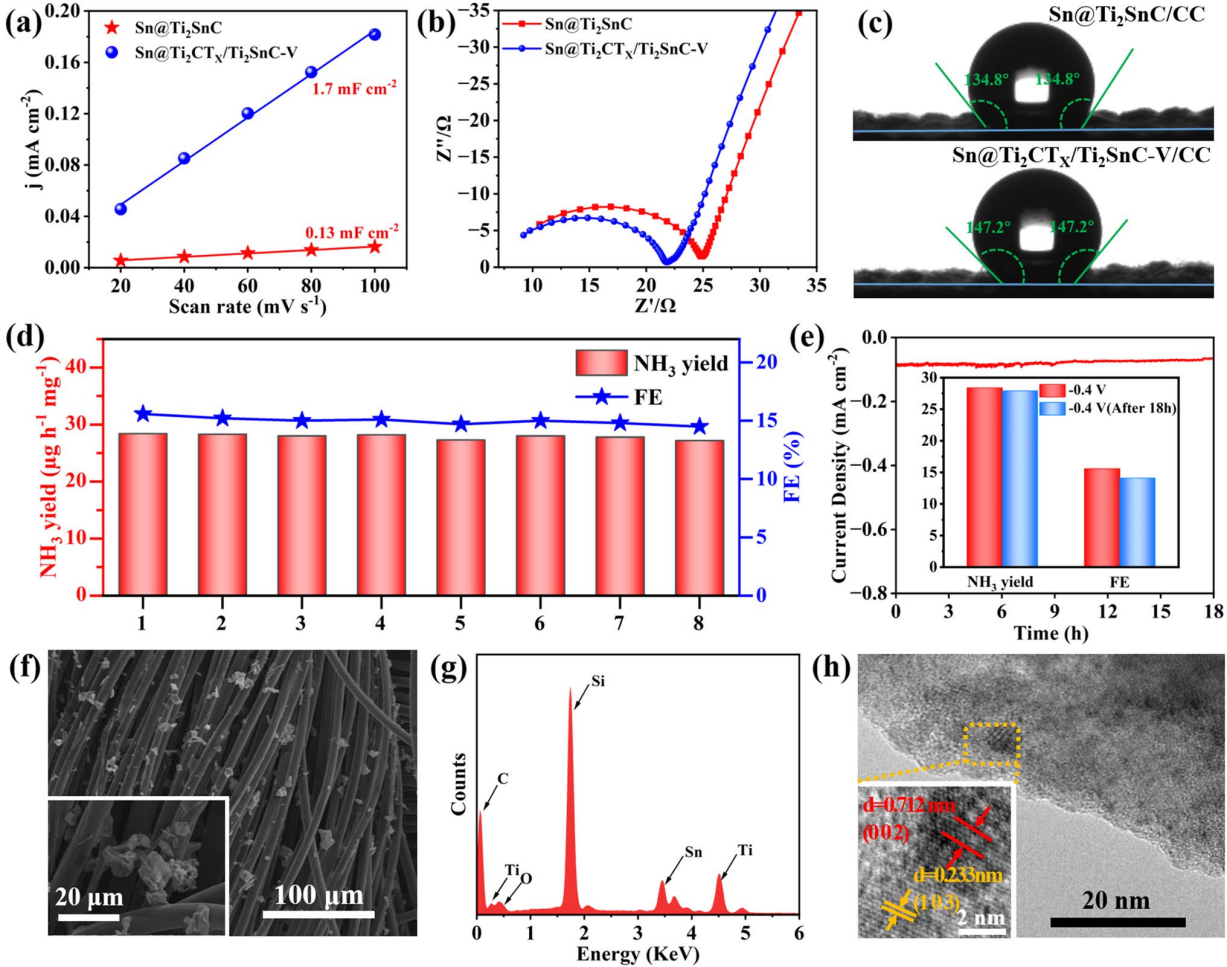
**a** C_dl_, **b** EIS, and **c** water droplet contact angle measurement of Sn@Ti_2_SnC/CC and Sn@Ti_2_CT_*X*_/Ti_2_SnC–V/CC. **d** Cycling tests of Sn@Ti_2_CT_*X*_/Ti_2_SnC–V and corresponding NH_3_ yields and FEs after each cycle. **e** Chronoamperometry test of Sn@Ti_2_CT_*X*_/Ti_2_SnC–V/CC at − 0.4 V (vs. RHE) with the inset demonstrates FEs and NH_3_ yields before and after 18 h durability test. **f** SEM, **g** EDS, **h** TEM and corresponding HRTEM images (inset) of Sn@Ti_2_CT_*X*_/Ti_2_SnC–V/CC after 18 h electrocatalysis

The excellent ENRR performances of the as-prepared Sn@Ti_2_CT_*X*_/Ti_2_SnC–V are mainly attributed to the following aspects. Firstly, Ti_2_CT_*X*_/Ti_2_SnC hybrid as an ideal support with large specific surface area, enables Sn highly confined on the surface of Ti_2_CT_*X*_/Ti_2_SnC heterostructure without agglomeration, thus exposing more active sites. Importantly, the Ti_2_CT_*X*_/Ti_2_SnC heterostructure endows the 3d orbitals of Sn atoms with lower electron density, which helps to strengthen the Sn-N bond for a better adsorption of N_2_ [6]. This highly dispersed Sn and Sn vacancies, as active centers for the ENRR, boost the adsorption and activation of N_2_, simultaneously suppress the HER [23]. For evaluating the electrochemically active surface area (ECSA), a series of cyclic voltammetry (CV, Fig. S13) measurements were performed to determine the double-layer capacitance (C_dl_) [68]. As shown in Fig. 4a, the C_dl_ of Sn@Ti_2_CT_*X*_/Ti_2_SnC–V is 1.7 mF cm^−2^, significantly higher than that of Sn@Ti_2_SnC (0.13 mF cm^−2^), indicating that Sn@Ti_2_CT_*X*_/Ti_2_SnC–V provides more catalytic active sites for ENRR. The increased ECSA of Sn@Ti_2_CT_*X*_/Ti_2_SnC–V can be attributed to the widening of interlayer distance, and the Ti_2_CT_*X*_/Ti_2_SnC substrate for preventing the aggregation of Sn. Secondly, Sn@Ti_2_CT_*X*_/Ti_2_SnC–V exhibits excellent electrical conductivity and lower transmission resistance, which is conducive to accelerating the whole process of ENRR. Electrochemical impedance spectroscopy (EIS) was performed to evaluate the interfacial reaction and electrode kinetics during ENRR process (Fig. 4b). The Sn@Ti_2_CT_*X*_/Ti_2_SnC–V exhibits a much smaller charge transfer resistance (*R*_ct_) of 21.6 Ω than that of Sn@Ti_2_SnC (24.8 Ω), which boosts the electron transportation from current collector to the active sites. This decrease in Rct is attributed to the increase in the MXene layer spacing [69] and the presence of the Ti_2_CT_*X*_/Ti_2_SnC heterostructure, in which the Ti_2_SnC phase can be effectively activated and the Ti_2_CT_*X*_ phase acts as a good electron conductor, connecting the Sn active sites of Ti_2_SnC into the electrochemical network [70]. Last but not least, according to the results of the contact angle test (Fig. 4c), both of two samples loaded on the carbon cloth are hydrophobic, that can effectively prevent water molecules from contacting with active sites for inhibiting HER, thus further accelerating the ENRR process [13, 71]. Overall, the factors mentioned above synergistically endow Sn@Ti_2_CT_*X*_/Ti_2_SnC–V with superior ENRR activity.

Stability is of importance as indexes to evaluate the performance of electrocatalysts, especially for practical applications. For this reason, cycling tests and long-term continuous potentiostatic electrolysis were carried out to evaluate the stability of the as-synthesized Sn@Ti_2_CT_*X*_/Ti_2_SnC–V electrode. As shown in Fig. 4d, no significant reduction in NH_3_ yields and FEs is observed during eight parallel tests at − 0.4 V (vs. RHE) in 0.1 M Na_2_SO_4_ for 2 h, indicating the high electrochemical stability of Sn@Ti_2_CT_*X*_/Ti_2_SnC–V toward ENRR in neutral media. In addition, the stable current density of 18 h long-term continuous potentiostatic electrolysis at − 0.4 V (vs. RHE) with 1.7% loss in NH_3_ yield and 9.4% loss in FE further demonstrates the remarkable electrochemical durability of Sn@Ti_2_CT_*X*_/Ti_2_SnC–V (Fig. 4e). Furthermore, the results of SEM (Fig. 4f), EDS (Fig. 4g) and TEM (Fig. 4h) of Sn@Ti_2_CT_*X*_/Ti_2_SnC–V after the ENRR tests confirm that the morphology and structure of the catalyst are almost unchanged after long term electrocatalysis, proving its robustness toward ENRR. This excellent durability of Sn@Ti_2_CT_*X*_/Ti_2_SnC–V is attributed to the existence of partial etched Ti_2_SnC MAX phase and O functional groups on the surface of Ti_2_CT_*X*_ MXene. The O terminals with strong electron absorption properties render Ti atoms in Ti_2_CT_*X*_ MXene with the highest oxidation state, thus boost the antioxidant capacity of Ti_2_CT_*X*_ MXene [57].

### PV-EC System

Another crucial factor for ENRR realizing large-scale green NH_3_ production is reducing the cost and energy consumption of this technique, which can realize by shortening the energy utilization path and avoiding using high-purity N_2_ as nitrogen source. Among various green electricity generation techniques, “solar farm” realized by photovoltaics (PV) panels is regarded as the most promising one due to the abundant and clean energy source of sunlight, the mature photoelectric conversion device, and the universality of this technique around the world [72]. In view of this, photovoltaic electrochemical (PV-EC) system represents an effective and sustainable way for green NH_3_ production, due to the combination of direct utilization of solar energy and the merits of electrocatalysis [73]. Therefore, we transfer our effort from laboratory experiments to outdoor investigations, which directly use solar panels as electricity source, air as nitrogen source, ultrapure water as proton source for the ENRR under environmental conditions.

**Fig. 5:**
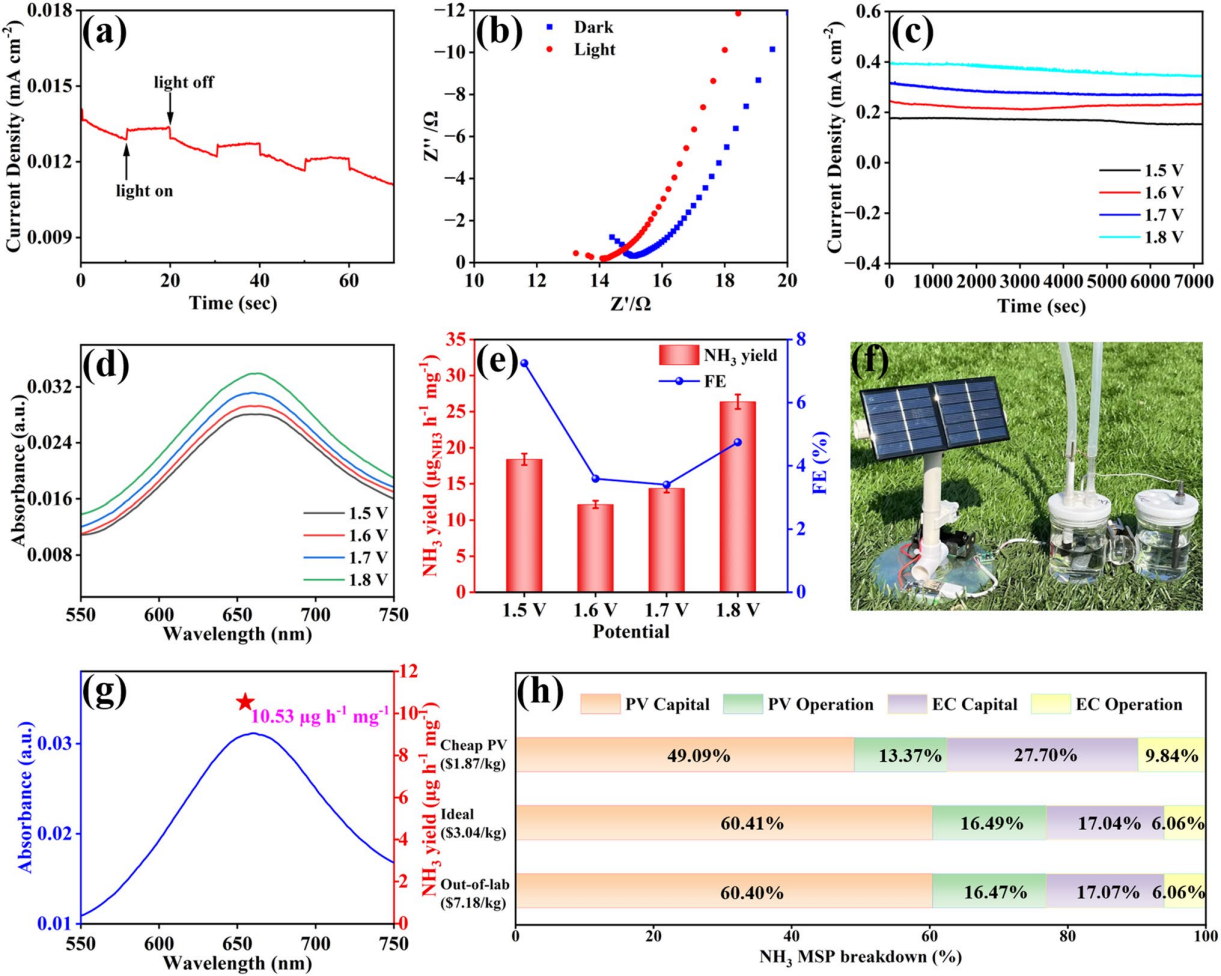
**a** Photocurrent response of Sn@Ti_2_CT_*X*_/Ti_2_SnC–V obtained on/off 150 W Xe light irradiation at the potential of 0 V in N_2_-saturated 0.1 M Na_2_SO_4_. **b** EIS of Sn@Ti_2_CT_*X*_/Ti_2_SnC–V in light and dark conditions. **c** Chronoamperometric measurements results under different potentials. **d** UV–Vis absorption spectra of the electrolytes after 2 h ENRR and corresponding **e** NH_3_ yields and FEs of Sn@Ti_2_CT_*X*_/Ti_2_SnC–V at different potentials in a two-electrode configuration. **f** Real picture of the PV-EC system. **g** UV–visible spectrum and NH_3_ yield (star location) after reaction for 2 h under the sun at 1.8 V. The techno-economic accounting and analysis. **h** A cost breakdown of the minimum selling price for renewable NH_3_

Firstly, photosensitivity of Sn@Ti_2_CT_*X*_/Ti_2_SnC–V was tested to investigate the effect of sunlight illumination on the ENRR process. As shown in Figs. 5a and S14, the photocurrent response of Sn@Ti_2_CT_*X*_/Ti_2_SnC–V measured at 0 V shows little difference on photocurrent between Xe light irradiation and dark, indicating the subtle influence of sunlight illumination on ENRR. In addition, as shown in the EIS (Fig. 5b), the transmission resistance of Sn@Ti_2_CT_*X*_/Ti_2_SnC–V under Xe lamp irradiation is smaller than that without irradiation, indicating that sunlight illumination can reduce the resistance of Sn@Ti_2_CT_*X*_/Ti_2_SnC–V and promote the charge transfer during ENRR. Then, for determining the optimal potential for NH_3_ production in the home-made electrocatalytic demonstrator outside the laboratory, we did comparison tests at potentials of 1.5, 1.6, 1.7 and 1.8 V, respectively, in a quartz H-type electrolytic cell filled with N_2_-saturated 0.1 M Na_2_SO_4_, of which Sn@Ti_2_CT_*X*_/Ti_2_SnC–V/CC was used as working electrode and a carbon rod as counter electrode. The Chronoamperometric measurements of Sn@Ti_2_CT_*X*_/Ti_2_SnC–V and the corresponding UV–Vis absorption spectra are shown in Figs. 5c, d and S15–S17. The optimal NH_3_ yield of 26.37 µg h^−1^ mg^−1^ obtained at 1.8 V (Fig. 5e), which is chosen as the applied potential. Finally, ENRR experiments were performed outside the laboratory by using a commercially available photovoltaic (PV) solar panel (0.55 W, 110 mA, 5 V) and a dual electrode configuration filled with 0.1 M Na_2_SO_4_ saturated with purified air (Figs. 5f and S18). Corresponding UV–visible absorption spectral curve is shown in Fig. 5g. Despite the presence of O_2_, this system achieved an optimal NH_3_ yield of 10.53 µg h^−1^ mg^−1^ in 1.8 V. Although this NH_3_ yield is only 40% of that obtained in ultra-high purity N_2_ saturated electrolyte, it does provide a practical, economical and carbon neutral way for green NH_3_ production.

In addition, a comparative techno-economic analysis of the PV-EC system was performed based on state-of-the-art demonstration for further investigating the economics of PV driven NH_3_ production, as depicted in Table S2. To achieve a reliable analysis, the annual solar radiation energy of Tibet, China (1875 kWh m^−2^) was used as the reference. Tables S3–S5 summarize the main assumptions for the economic analysis. In order to accommodate independent sustainable farms, it is assumed that the NH_3_ yield of PV-EC module is 1000 kg/year. Based on the NH_3_ yield obtained outside the laboratory (10.53 µg h^−1^ mg^−1^), the calculated minimum selling price (MSP) of NH_3_ is $7.18 kg^−1^. The cost details of NH_3_ MSP are shown in Fig. 5h and Table S6. PV module with its capital and operational costs accounting for 61% and 16% of the total cost act as the major cost driver. EC module with the capital and operational costs accounting for 17% and 6% of total cost is responsible for the second largest cost driver. Assuming that N_2_ as the nitrogen source in two-electrode system is the ideal scenario, when the number of photovoltaic modules sharply reduces from 139 to 59, and the number of electrochemical components reduces from 4380 to 2040. Therefore, the MSP of NH_3_ is greatly decreased to $3.04 kg^−1^ (Fig. 5h and Table S7). Assuming that the cost of PV modules is reduced to 50% of its original, the MSP of NH_3_ further reduces to $1.87 kg^−1^ (Fig. 5h and Table S8). If take the by-product of H_2_ into consideration, which will compensate for the cost, the MSP will achieve a lower price. These results demonstrate that the PV-EC module has a great prospect for industrialization ($0.5 kg^−1^) in terms of cost price. According to the global horizontal irradiation map (Fig. S19) and the photovoltaic development potential map with the red color shows the potential (Fig. S20), this PV-EC system has potential applications in more than half of the world’s land areas, and it also works in the ocean. The photovoltaic driven ENRR would be a cost-effective and sustainable strategy for efficient NH_3_ production.

## Conclusions

In summary, Sn@Ti_2_CT_*X*_/Ti_2_SnC heterostructure with abundant Sn vacancies was successfully prepared by controlled etching of Sn@Ti_2_SnC MAX phase. The highly dispersed Sn atoms throughout the Ti_2_CT_*X*_/Ti_2_SnC heterostructure provide adequate active sites for adsorption and activation of N_2_, simultaneously, the Sn vacancies between layers accelerate the charge transfer rate and reduce the adsorption energetics of the intermediates, thereby boosting the ENRR activity. Meanwhile, Ti_2_CT_*X*_/Ti_2_SnC heterostructure realize the balance of providing large active surface area and accelerating the stability. Therefore, Sn@Ti_2_CT_*X*_/Ti_2_SnC demonstrates a striking ENRR performance (NH_3_ yield: 28.4 µg h^−1^ mg_cat_^−1^, FE: 15.57%) at − 0.4 V versus RHE with an excellent durability up to 18 h in 0.1 M Na_2_SO_4_. In addition, a PV-EC modular demonstrator based on Sn@Ti_2_CT_*X*_/Ti_2_SnC–V electrode with a maximum ammonia productivity of 10.53 µg h^−1^ mg^−1^ in neutral electrolyte is proposed, which can successfully synthesize green ammonia from solar energy, ultrapure water and nitrogen form air. The systematic techno-economic analysis proves that this strategy is economic feasible and will open up a new direction for ammonia production beyond the laboratory in the future.

## Supplementary Information

Below is the link to the electronic supplementary material.

Supplementary file1 (DOCX 2535 KB)
