# Supplementary material for: Enhancing Green Ammonia Electrosynthesis Through Tuning Sn Vacancies in Sn-Based MXene/MAX Hybrids
Source: Nanomicro Lett. 2024 Jan 16;16:89. doi: 10.1007/s40820-023-01303-2 (PMC10792155; doi:10.1007/s40820-023-01303-2)
Supplement: Supplementary file 1 — Supplementary file1 (DOCX 2535 KB) [file 40820_2023_1303_MOESM1_ESM.docx]

Supporting Information

**Enhancing Green Ammonia Electrosynthesis through Tuning Sn**

**Vacancies in Sn-Based MXene/MAX Hybrids**

Xinyu Dai,^1,†^ Zhen-Yi Du,^2,†^ Ying Sun,^1,^* Ping Chen,^3^ Xiaoguang Duan,^4^ Junjun Zhang,^5^ Hui Li,^6^ Yang Fu,^6^ Baohua Jia,^6^ Lei Zhang,^7^ Wenhui Fang,^8^ Jieshan Qiu,^8,^* Tianyi Ma^6,^*

^1^Institute of Clean Energy Chemistry, Key Laboratory for Green Synthesis and Preparative Chemistry of Advanced Materials of Liaoning Province, College of Chemistry, Liaoning University, Shenyang 110036, P. R. China.

^2^State Key Laboratory of Clean and Efficient Coal Utilization, Taiyuan University of Technology, Taiyuan 030024, P. R. China.

^3^School of Chemistry and Chemical Engineering, Anhui University, Hefei 230601, P. R. China.

^4^School of Chemical Engineering, The University of Adelaide, Adelaide, SA 5005, Australia.

^5^State Key Laboratory of High-efficiency Utilization of Coal and Green Chemical Engineering, College of Chemistry & Chemical Engineering, Ningxia University, Yinchuan 750021, Ningxia, P. R. China.

^6^School of Science, RMIT University, Melbourne, VIC 3000, Australia.

^7^School of Chemistry and Chemical Engineering, Guangdong Provincial Key Laboratory of Advanced Energy Storage Materials, South China University of Technology, Guangzhou 510640, China.

^8^College of Chemical Engineering, State Key Laboratory of Chemical Resource Engineering, Beijing University of Chemical Technology, Beijing 100029, P. R. China.

† Xinyu Dai and Zhen-Yi Du contributed equally to this work.

*Corresponding author. E-mail: yingsun@lnu.edu.cn; qiujs@mail.buct.edu.cn; tianyi.ma@rmit.edu.au.

**S1 Experiment Details**

**Material:** Ti powder (99.8%, 300 mesh), Sn powder (99.5%, 200 mesh) and graphite (99.95%, 500 mesh) were purchased from Maclin Biochemical Technology Co., LTD. Nafion (5% wt%) was purchased from Shanghai Hesen Electric Co., LTD. Concentrated hydrochloric acid (HCl, 36 wt.%), concentrated sulfuric acid (H_2_SO_4_), hydrofluoric acid (HF, 40 wt.%), sodium hypochlorite solution (NaClO, effective chlorine ≥5.0%), ammonium chloride (NH_4_Cl, ≥99.99%), anhydrous sodium sulfate (Na_2_SO_4_, AR), potassium hydroxide (KOH, AR), potassium bicarbonate (KHCO_3_, AR), ethanol (C_2_H_5_OH 99.5%), sodium salicylate (C_7_H_5_O_3_Na, AR), sodium nitroprusside dihydrate (Na_2_Fe(CN)_5_NO·2H_2_O, ≥98.0%), sodium potassium tartrate (NaKC_4_H_4_O_6_·4H_2_O, AR), aqueous hydrogen peroxide (H_2_O_2_, 30.0%) from Sinopharm Chemical Reagent Co., LTD. All reagents are used as received without further purification. Nafion 117 membranes (DuPont) are purchased from fuel cell stores. All ultrapure water used in the experiments was purified by the Millipore system (Millipore, 18.2 MΩ·cm). High purity N_2_ gas (≥99.999%) and Ar gas (≥99.999%) are purchased from Shenyang Zhaote Special Gas Co., LTD.

**Electrochemical measurements:** Nafion membranes (Nafion 117) were thermally treated at 80 ℃ for 1 h in 5% H_2_O_2_, 0.5 M H_2_SO_4_ and ultrapure water, respectively. After rinsing thoroughly with ultrapure water, the membranes were immersed in ultrapure water for future use. All experimental data for N_2_ electroreduction (ENRR) were collected at CHI-760E electrochemical workstation. An H-type electrolytic cell separated by Nafion 117 was used as the reaction device. The catalyst-loaded carbon cloth was employed as the working electrode, Ag/AgCl electrode (filled with 3.5 M KCl solution) as the reference electrode, and a carbon rod as the counter electrode. The volume of the electrolyte in the anode and cathode chamber is 70 mL for each. According to Nernst Eq. S1, all potentials reported in this work were calibrated to reversible hydrogen electrode (RHE):

E_RHE_ = E_Ag/AgCl_ + 0.059 × pH + 0.205 (S1)

Before the electrochemical tests, the Na_2_SO_4_ electrolyte (0.1 M) was bubbled with high-purity N_2_ or Ar for half an hour to expel the original air. The concentration of NH_3_ produced by ENRR procedure is determined by the indophenol blue method and the ammonia gas sensitive electrode method. The concentration of hydrazine in the electrolyte was quantified by Watt and Chrisp methods.

**Ammonia quantification by indophenol blue method:** The quantity of the produced NH_3_ was determined by indophenol blue method. Briefly, 4 mL of the electrolyte to be tested was added successively into 4 mL of coloring solution (1 M KOH solution containing 5 wt % sodium salicylate and 5 wt % potassium sodium tartrate), 2 mL of oxidizing agent (0.05 M NaClO solution) and 0.4 mL sodium nitroferricyanide solution (1 wt %), after mixing and standing for 1 h away from light. Then the absorption spectra were measured by UV-vis spectrophotometer. The formation of indophenol blue was determined by absorbance at λ = 655 nm.

**Ammonia quantification by ammonia-sensitive selecting electrode method:** First, a series of standard ammonia solutions (0.1, 0.2, 0.3, 0.4, and 0.5 µg mL^-1^ in 0.1 M Na_2_SO_4_) were prepared from the stock solution (1000 ppm ammonia as the nitrogen standard) for calibration. Ionic strength adjuster (ISA) was used to provide constant background ionic strength and adjust solution pH. ISA must be added to all samples and standards immediately before measurement to prevent ammonia loss. 5 mL standard or sample should be added with 0.2 mL ISA and thoroughly stirred, followed by 5 mL of 1 M KOH solution. The test should be started by quickly plunging the electrode below the liquid level, taking care not to leave any air bubbles.

**Hydrazine Determination:** The content of hydrazine, a possible byproduct in electrolytes, was analyzed by Watt and Chrisp method. The coloring solution for hydrazine was prepared by dissolving 2.0 g p-(dimethylamino-benzaldehyde) in the mixture of 10 mL concentrated hydrochloric acid and 100 mL ethanol. After adding 5 mL electrolyte into 5 mL coloring solution, the adsorption spectrum was obtained by using UV-vis spectrophotometer after 20 min. The concentration of hydrazine was determined using the absorbance signal at λ = 455 nm.

**Calculation of NH_3_ yield and Faradaic efficiency:** The ammonia formation rate was determined using the following Eq. S2:

NH_3_ yield= (c × *V*)/(t × *m*) (S2)

where *c* is the measured NH_3_ concentration, *V* is the volume of the electrolyte, *t* is the electrochemical reaction time, and *m* is the mass of the catalyst.

The Faradaic efficiency was calculated according to Eq. S3 as follows:

FE= (3× *F* × *c* × *V*)/(17 × *Q*) (S3)

where *F* is the Faraday constant, 96485 C/mol, *c* is the measured NH_3_ concentration, *V* is the volume of the electrolyte, and *Q* is the total charge used for the electrodes.

**Measurement of Double layer capacitance:** Cyclic Voltammetry (CV) was measured under the potential window of 0.32 V ~ 0.42 V versus Ag/AgCl (vs. Ag/AgCl), and the scanning rates were 20, 40, 60, 80 and 100 mV, respectively. By plotting (j) against the sweep rate at 0.37 V vs. Ag/AgCl, the slope value can be calculated as the double layer capacitance (C_dl_).

**The techno-economical accounting (TEA) of photovoltaic electrochemical (PV-EC) system:** The integrated photovoltaic electrochemical system is composed of multiple photovoltaic modules and electrochemical modules in parallel. It is worth noting that the total DC output power of the photovoltaic module needs to be equal to the total power required by the electrochemical module. In this TEA, the non-use of batteries resulted in a capacity factor of 21.34%. The simulation of the system is based on indoor and outdoor ENRR experimental data, and the potential of the three-electrode system (-0.4 V vs. RHE) is converted to the potential of the two-electrode system to achieve similar ammonia production (1.8 V).

Table S2 summarizes the design of the electrochemical (EC) module, consisting of an anode and a cathode, carbon paper loaded with 1.0 g catalyst and bare carbon paper, respectively. Both electrodes placed in 0.1 M Na_2_SO_4_ solution and providing 12.25 W of power at 2.5 V and 4.9 A. Another important component is the photovoltaic (PV) module, which we used a monocrystalline silicon solar panel model SR-54M425NHL Pro (Table S3). Tibet, with its better solar energy resources and high capacity factor, was chosen as the site for this analysis. The photovoltaic module has an annual output of 800 kWh. The efficiency of the EC module can be measured by the Eq. S3.

The economic performance of PV-EC modules can be measured by the minimum selling price (MSP) of ammonia, calculated as following:

MSP_NH3_= (CAPEX × CRF + OPEX)/m_NH3_ (S4)

where CAPEX, the total capital expenditure, includes the capital expenditure of PV modules and EV modules; CRF is the return on capital on annual capital. Assuming an 8% interest rate and a 25-year product life, the CRF is 0.094; OPEX is the annual operation and maintenance cost; m_NH3_ refers to the mass of annual ammonia production, assuming that the average annual ammonia production is 1000 kg.

**Characterization**

The crystalline phase of the specimens was analyzed by the X-ray diffractometer (Bruker D8 Advance). The morphology and lattice fringe information were analyzed by SEM (Hitachi SU-8010) and TEM (JEM-2100). The elements of the samples were analyzed by X-ray photoelectron spectrometer (Escalab 250Xi). The presence of vacancies was detected using a paramagnetic resonance spectrometer (Bruker EMXnano). The hydrophobicity and hydrophilicity of the materials were analyzed by a contact Angle tester (DSA100). Ultraviolet-visible data were obtained using a Shimazu ultraviolet-visible spectrophotometer (UV-2600).

**S2 Supplementary Figures and Tables**


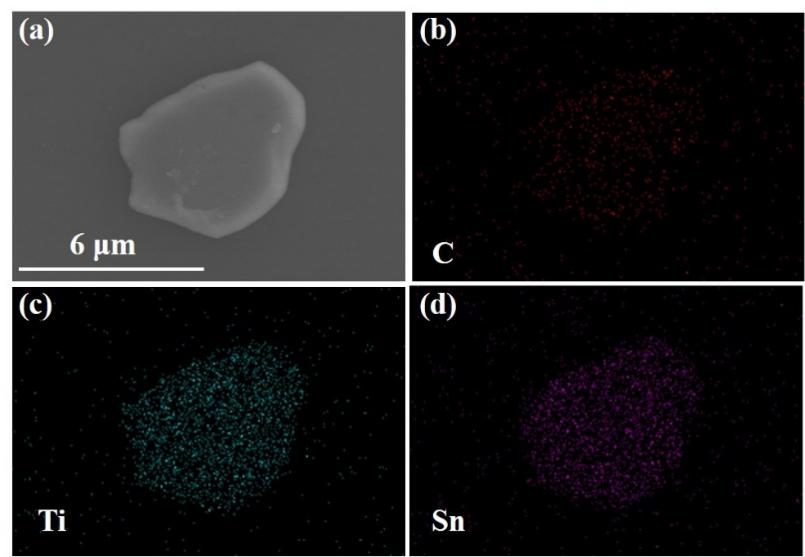


**Fig. S1** SEM images and the corresponding elemental mappings of Sn@Ti_2_SnC


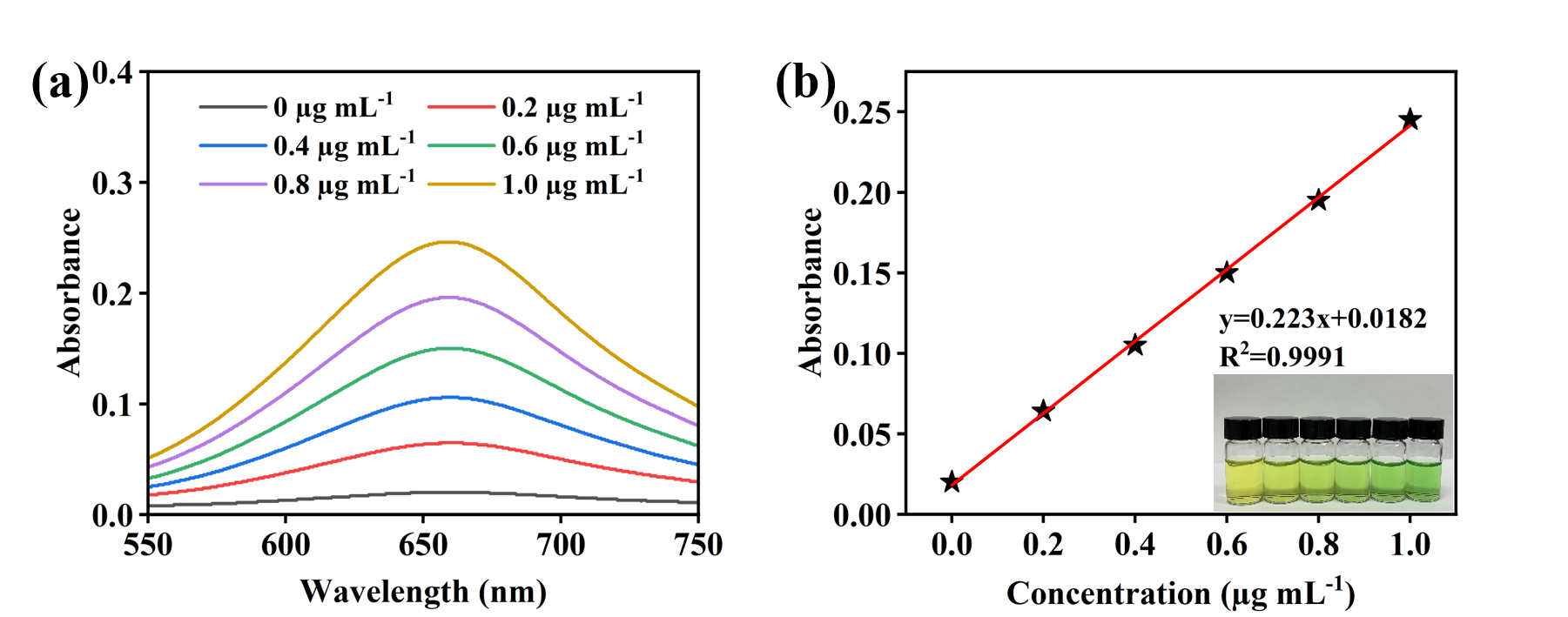


**Fig. S2** **a** UV-vis spectra for indophenol assays with NH_4_^+^ ions after incubation for 2 h in 0.1 M Na_2_SO_4_. **b** Calibration curve for NH_4_^+^ concentration in 0.1 M Na_2_SO_4_ to estimate NH_3_ yield. The inner illustration is the chromogenic reaction of indoxol indicator with NH_4_^+^ ion


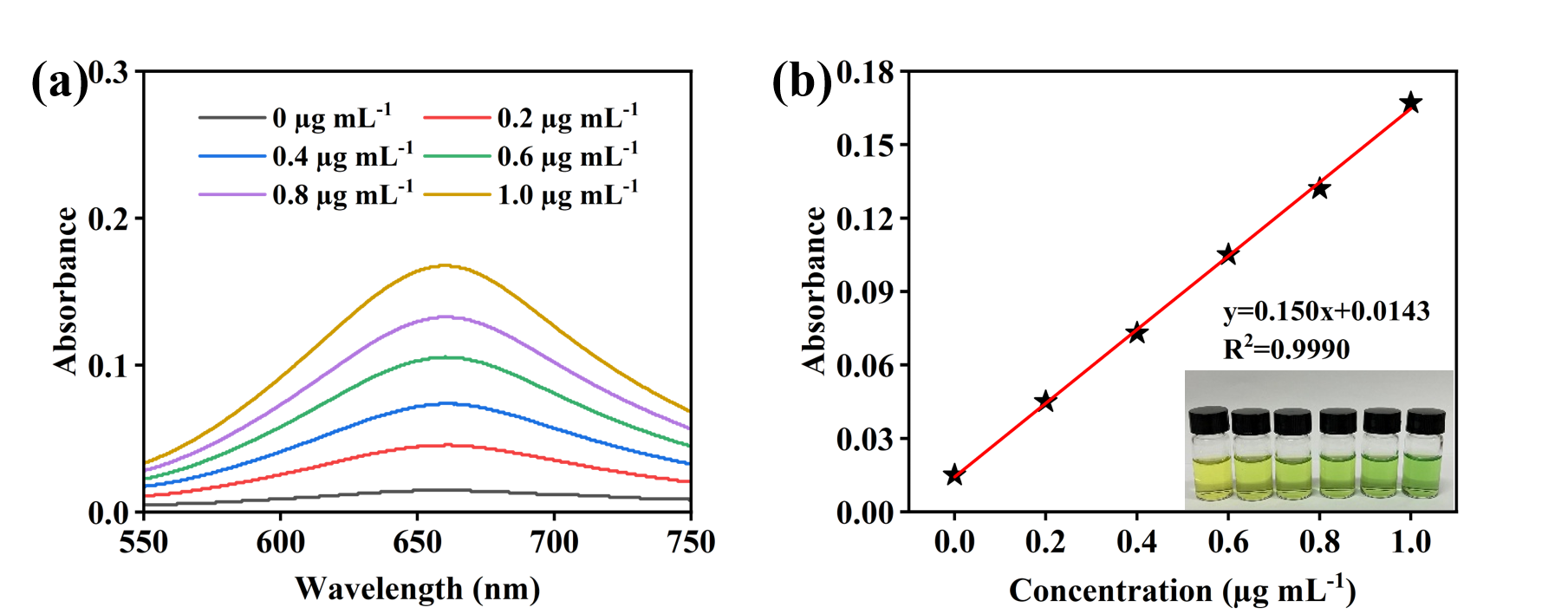


**Fig. S3** **a** UV-vis spectra for indophenol assays with NH_4_^+^ ions after incubation for 2 h in 0.01 M H_2_SO_4_. **b** Calibration curve for NH_4_^+^ concentration in 0.01 M H_2_SO_4_ to estimate NH_3_ yield. The inner illustration is the chromogenic reaction of indoxol indicator with NH_4_^+^ ion


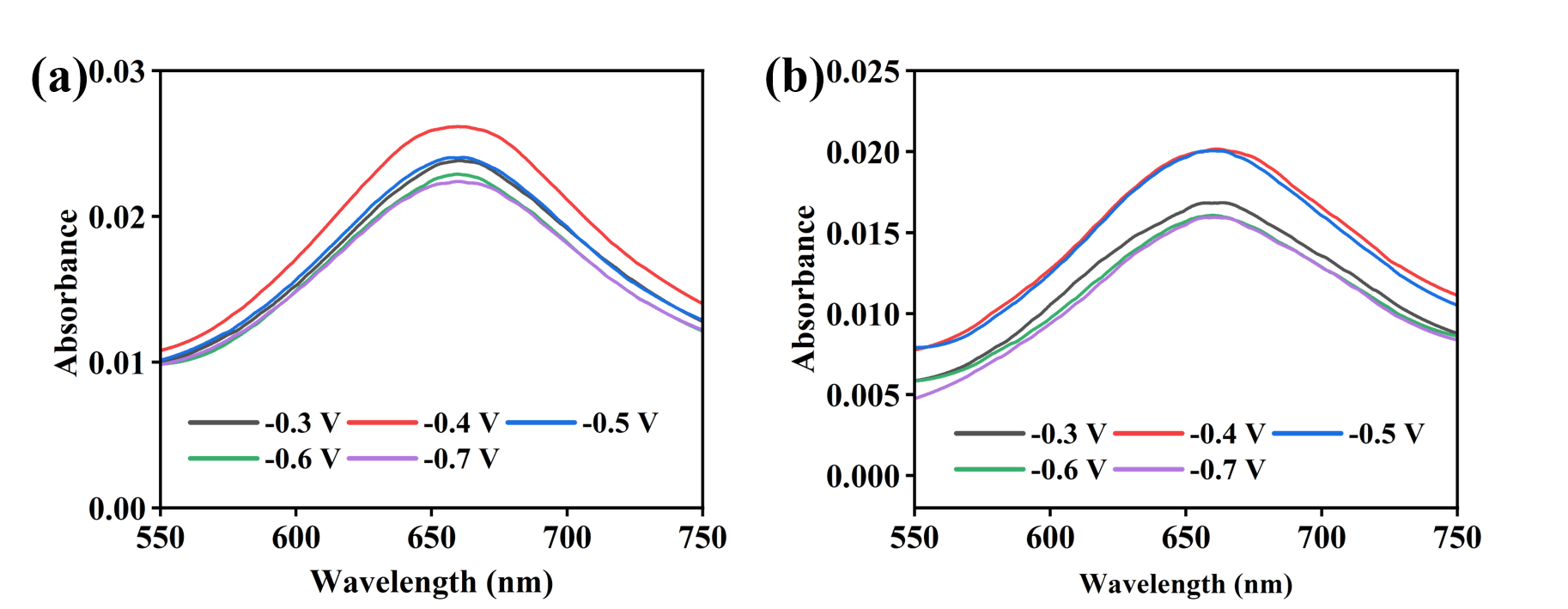


**Fig. S4** UV-vis spectra of Sn@Ti_2_CT_X_/Ti_2_SnC-V at different potentials of **a** 0.1 M Na_2_SO_4_ electrolyte and **b** 0.01 M H_2_SO_4_ for 2 h


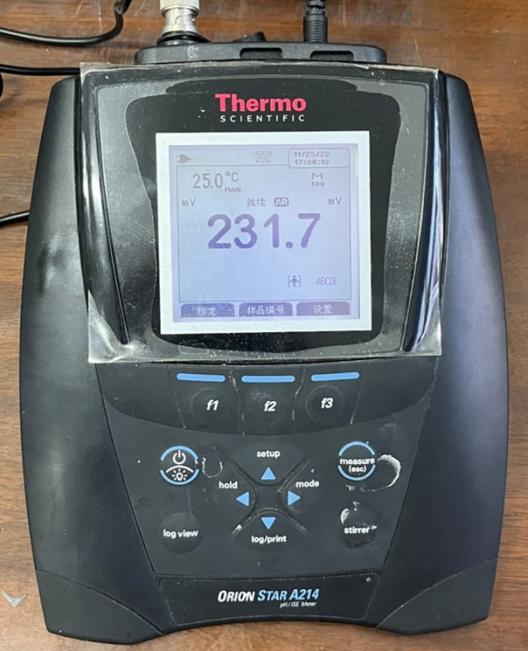


**Fig. S5** Photograph of ammonia-sensitive testing instrument


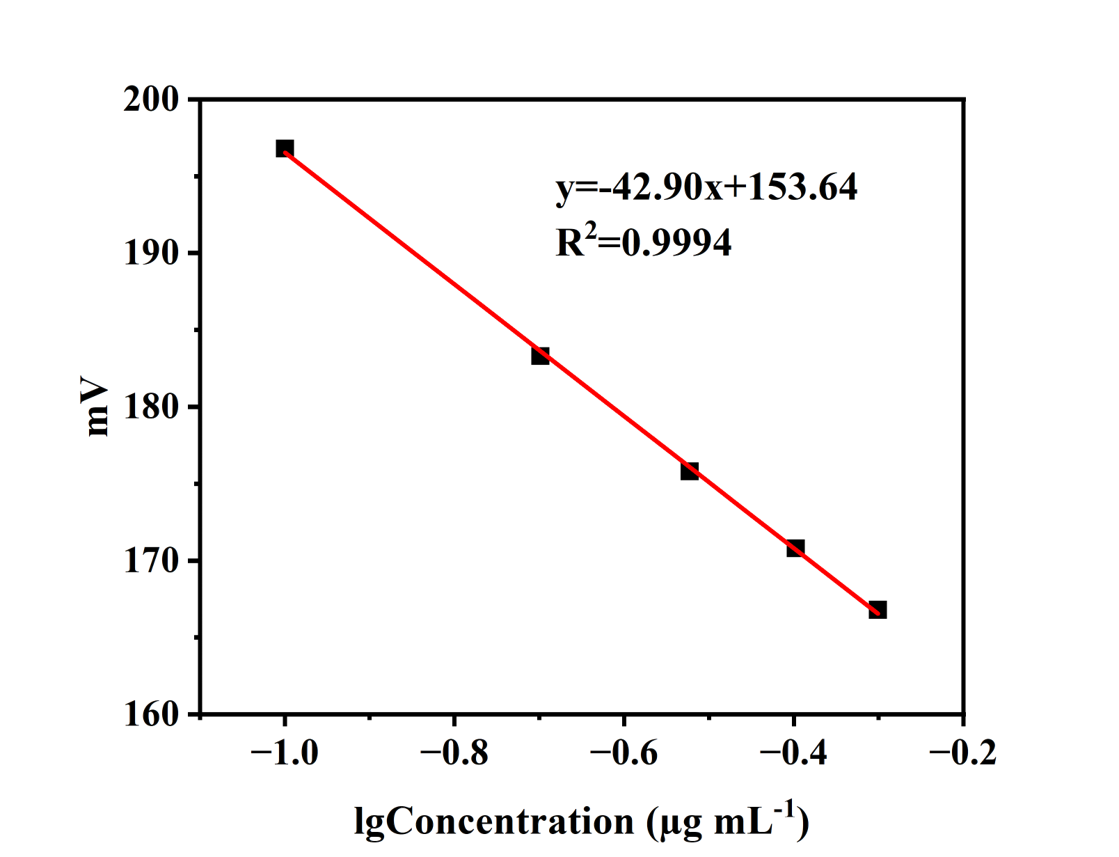


**Fig. S6** Calibration of NH_3_ with ammonia-sensitive testing instrument in 0.1 M Na_2_SO_4_


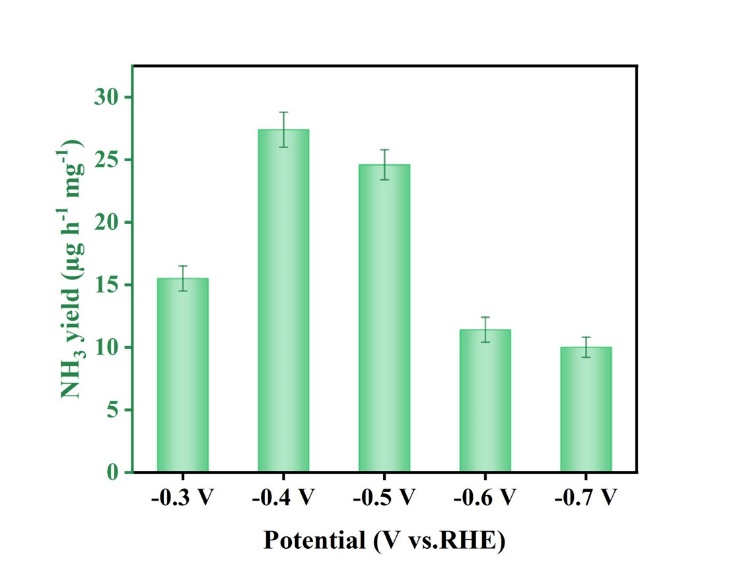


**Fig. S7** NH_3_ yields and FEs of Sn@Ti_2_CT_X_/Ti_2_SnC-V with ammonia-sensitive testing instrument in 0.1 M Na_2_SO_4_


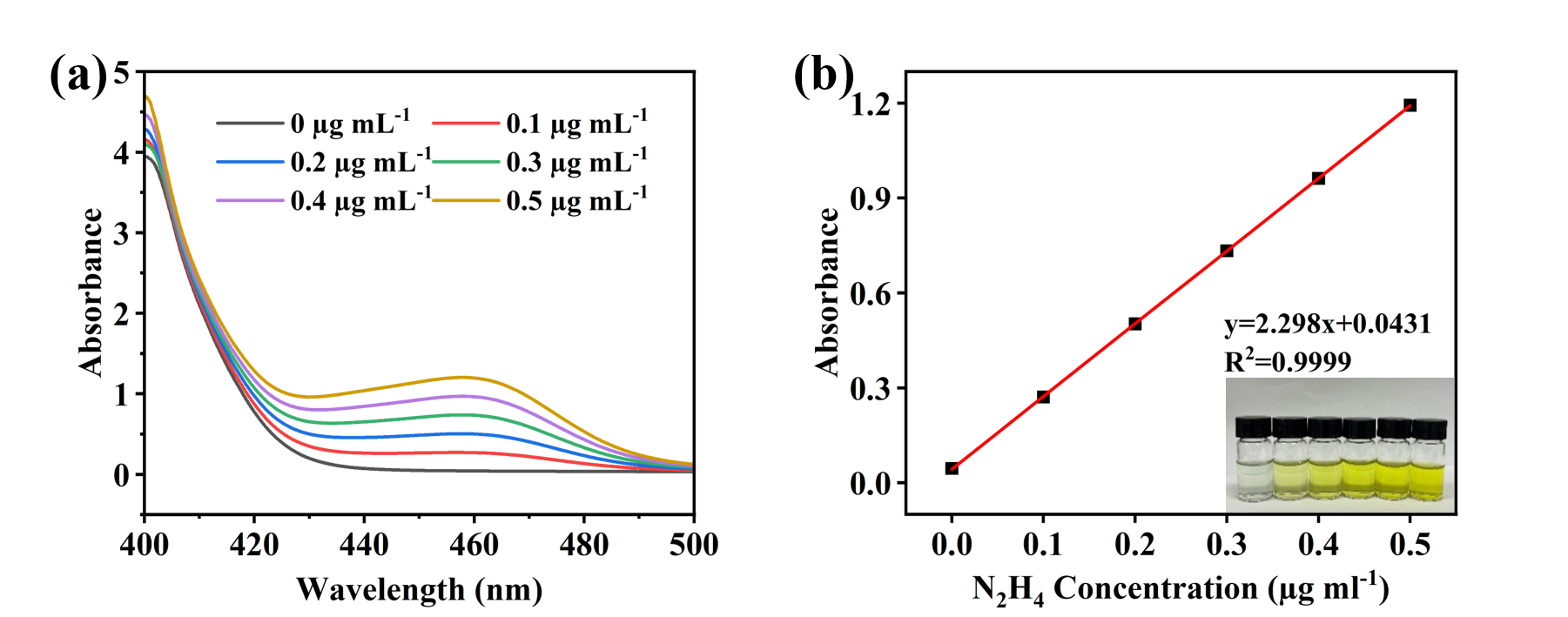


**Fig. S8** **a** UV-vis curves for various N_2_H_4_·H_2_O concentrations after incubation for 10 min at room temperature and **b** Calibration curve used for the estimation of the N_2_H_4_·H_2_O concentration. The inset of **b** shows the chromogenic reaction of the indicator with N_2_H_4_·H_2_O


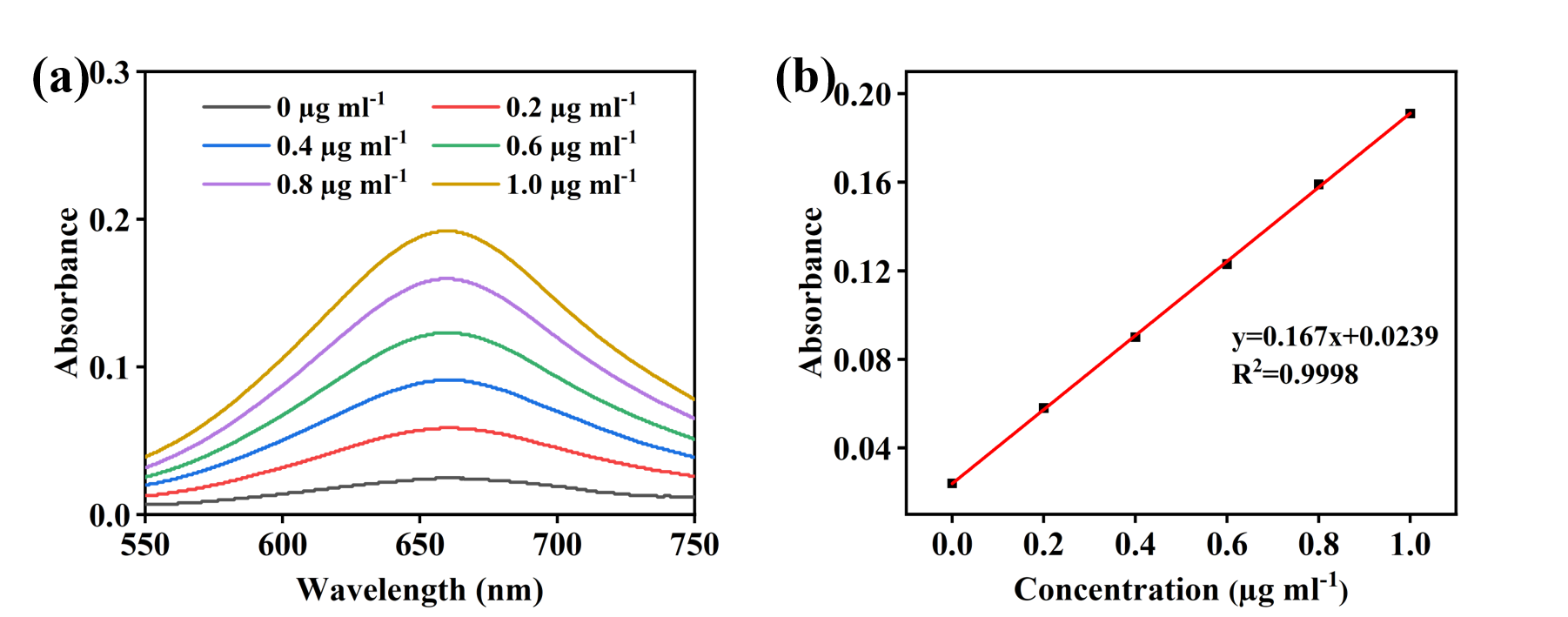


**Fig. S9 a** UV-vis spectra for indophenol assays with NH_4_^+^ ions after incubation for 2 h in 0.1 M HCl. **b** Calibration curve for NH_4_^+^ concentration in 0.1 M HCl to estimate NH_3_ yield


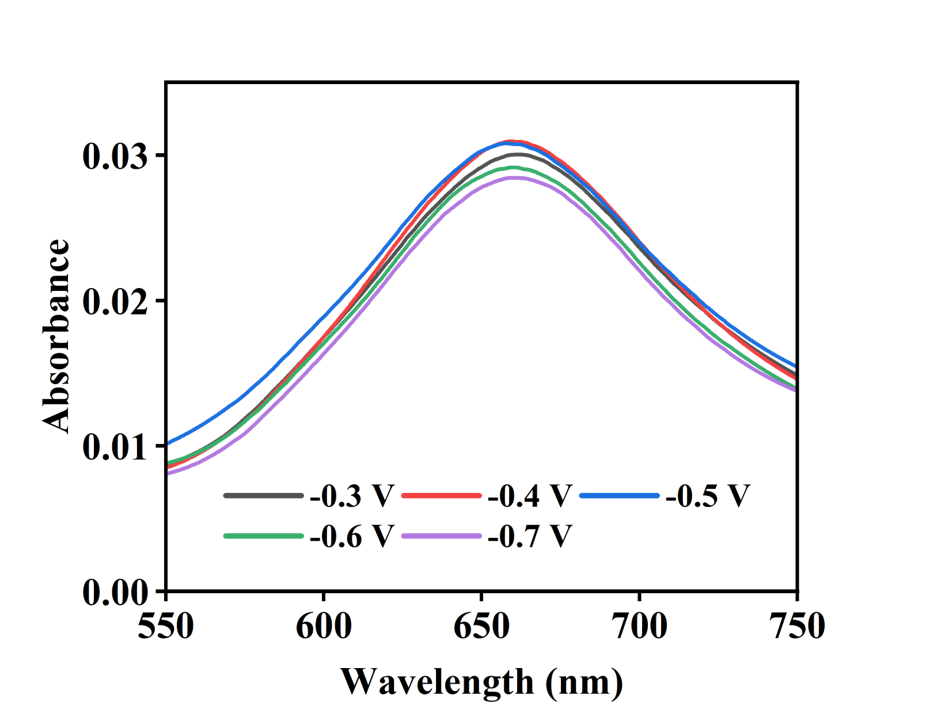


**Fig. S10** UV-vis spectra of Sn@Ti_2_CT_X_/Ti_2_SnC-V at different potentials of 0.1 M HCl for 2 h


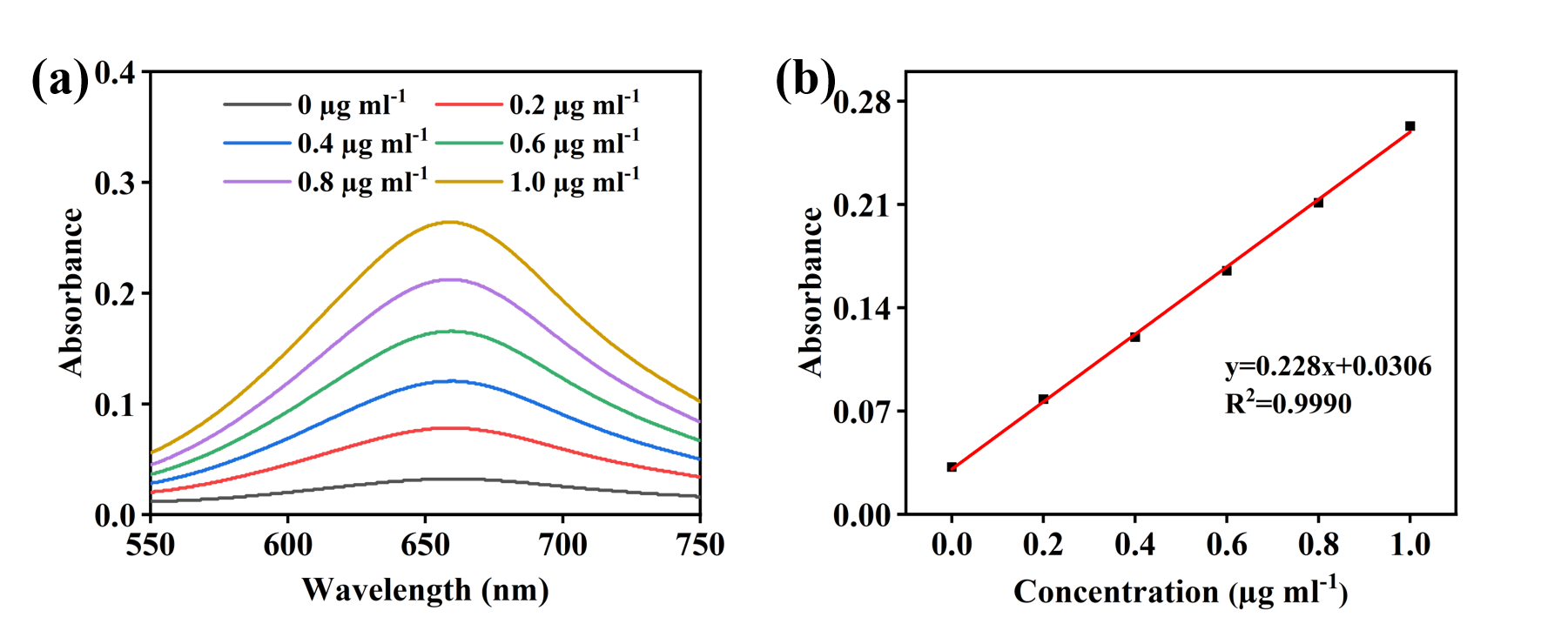


**Fig. S11 a** UV-vis spectra for indophenol assays with NH_4_^+^ ions after incubation for 2 h in 0.1 M KHCO_3_. **b** Calibration curve for NH_4_^+^ concentration in 0.1 M KHCO_3_ to estimate NH_3_ yield


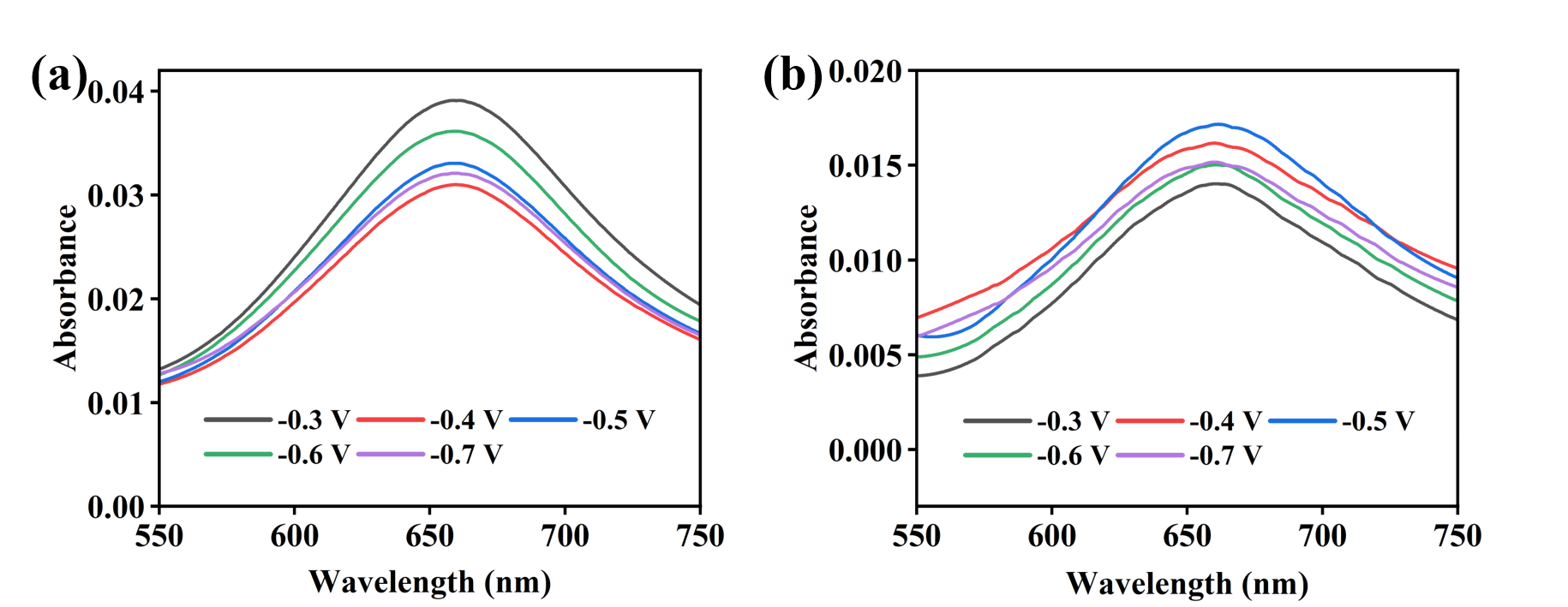


**Fig. S12** UV-vis spectra of Sn@Ti_2_CT_X_/Ti_2_SnC-V at different potentials of **a** 0.1 M KHCO_3_ and **b** 0.01 M H_2_SO_4_ for 2 h


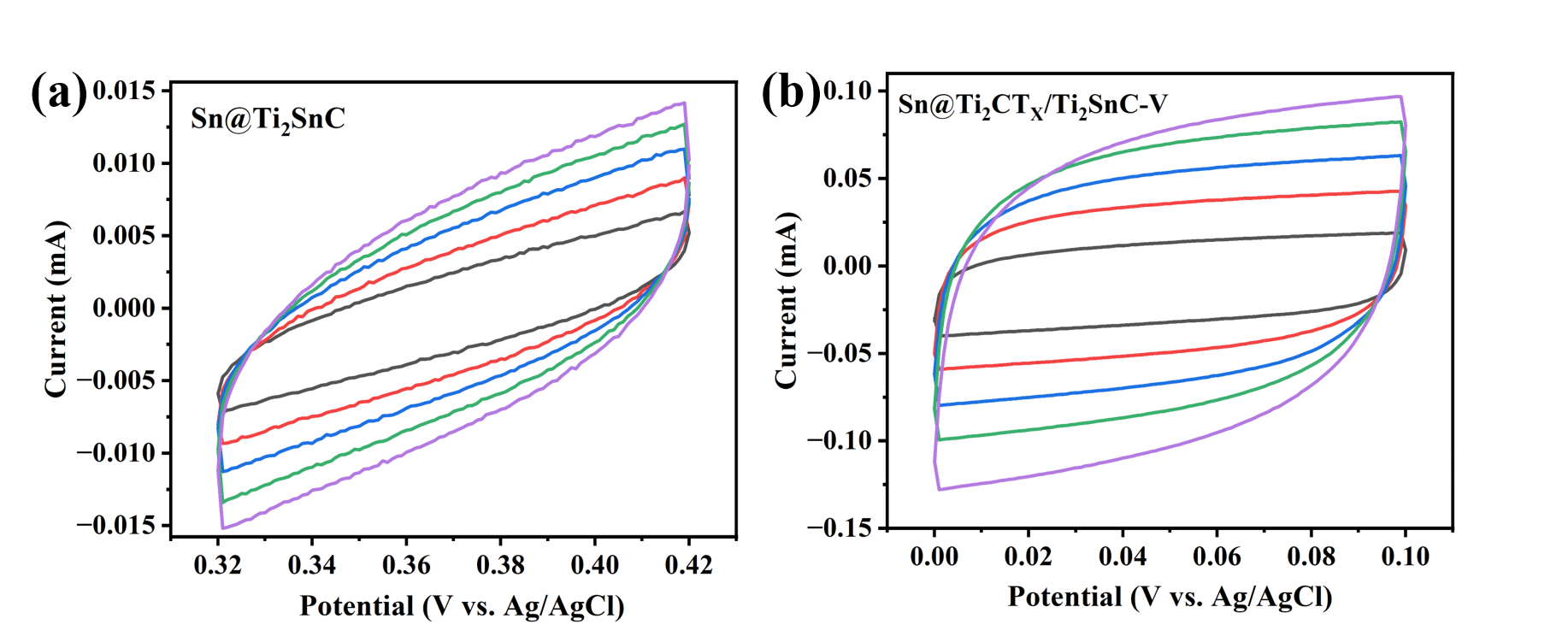


**Fig. S13** CV curves of **a** Sn@Ti_2_SnC, **b** Sn@Ti_2_CT_X_/Ti_2_SnC-V


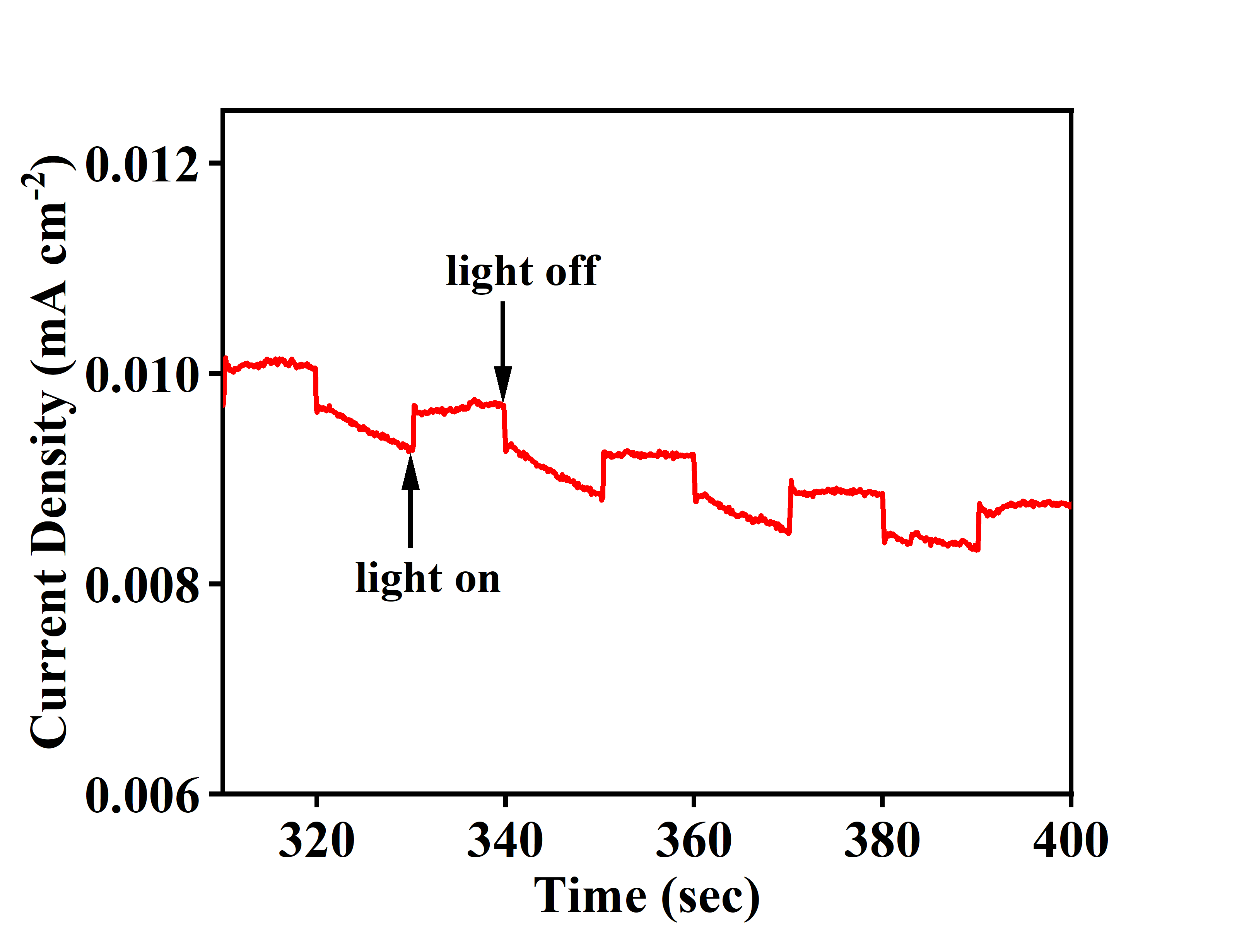


**Fig. S14** Photocurrent response of Sn@Ti_2_CT_X_/Ti_2_SnC-V obtained on/off 150 W Xe light irradiation after 310s at the potential of 0 V in N_2_-saturated 0.1 M Na_2_SO_4_


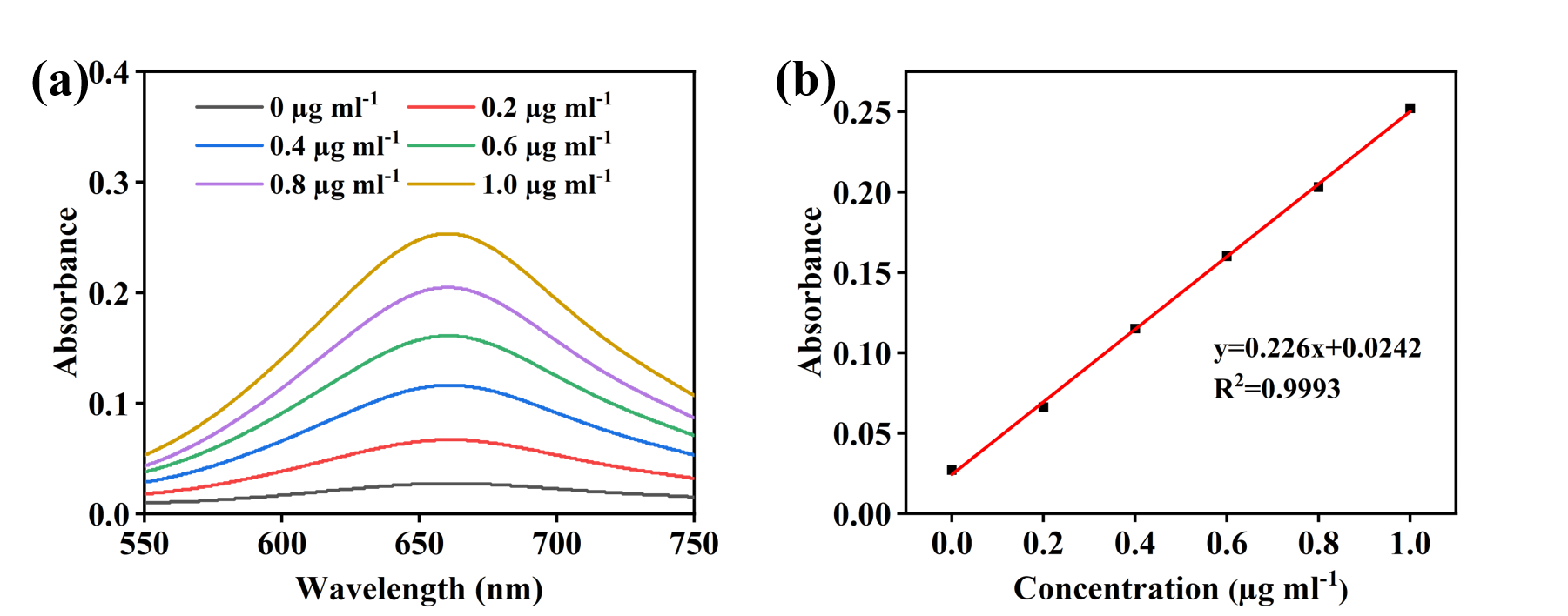


**Fig. S15** **a** UV-vis spectra of for indophenol assays with NH_4_^+^ ions after incubation for 2 h in 0.1 M Na_2_SO_4_. **b** Calibration curve for NH_4_^+^ concentration in 0.1 M Na_2_SO_4_ to estimate NH_3_ yield


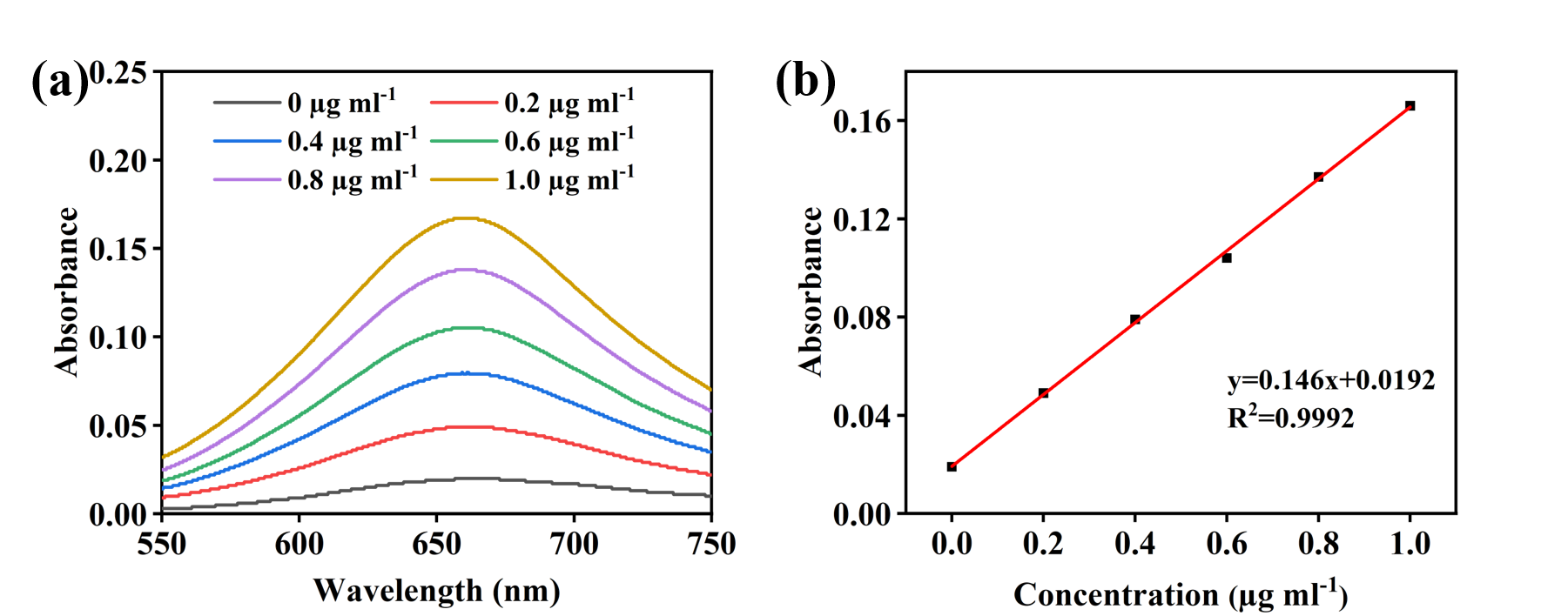


**Fig. S16** **a** UV-vis spectra for indophenol assays with NH_4_^+^ ions after incubation for 2 h in 0.01 M H_2_SO_4_. **b** Calibration curve for NH_4_^+^ concentration in 0.01 M H_2_SO_4_ to estimate NH_3_ yield


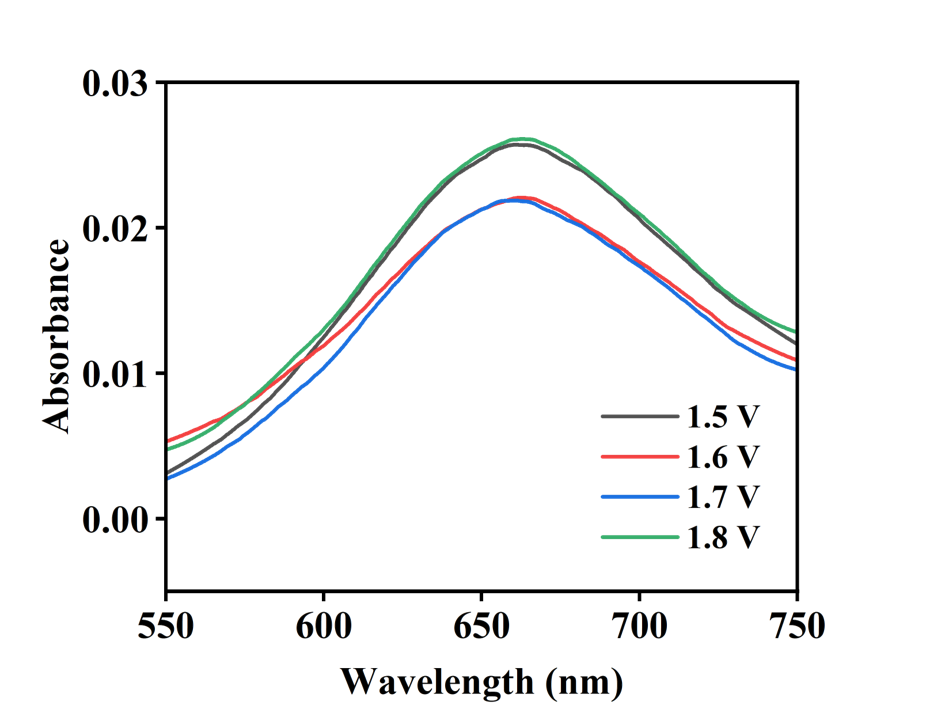


**Fig. S17** UV-vis spectra of Sn@Ti_2_CT_X_/Ti_2_SnC-V at different potentials in 0.01 M H_2_SO_4_ at double electrodes for 2 h


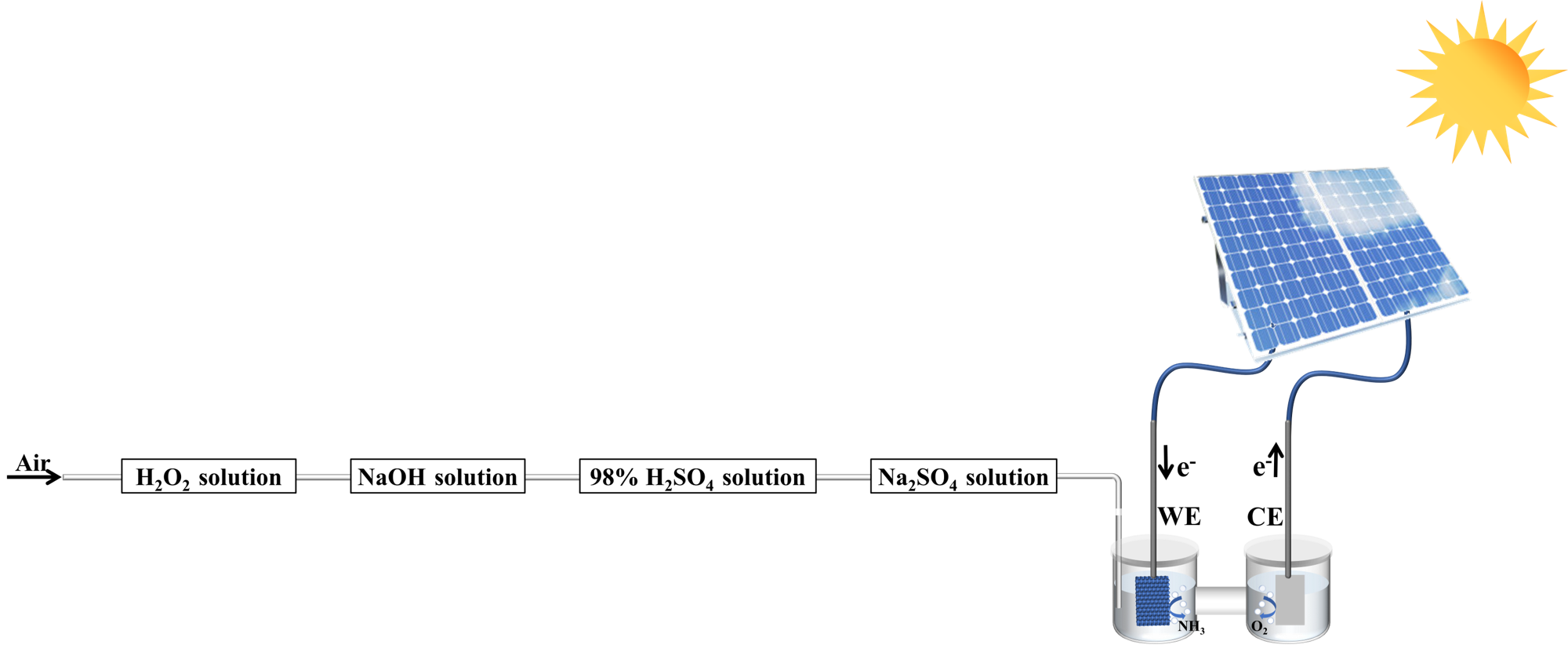


**Fig. S18** Gas purification to remove any N contamination in the atmosphere


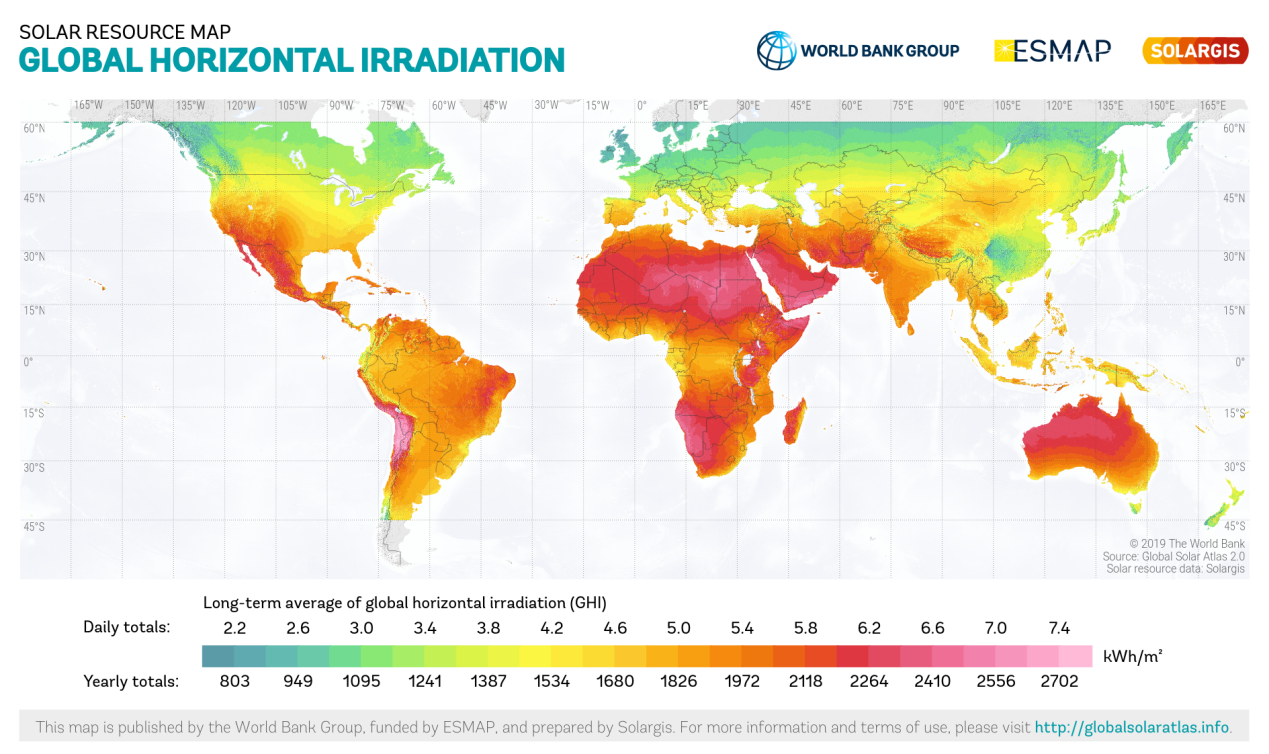


**Fig. S19** The map of global horizontal irradiation [S[1](#_ENREF_1_1)]


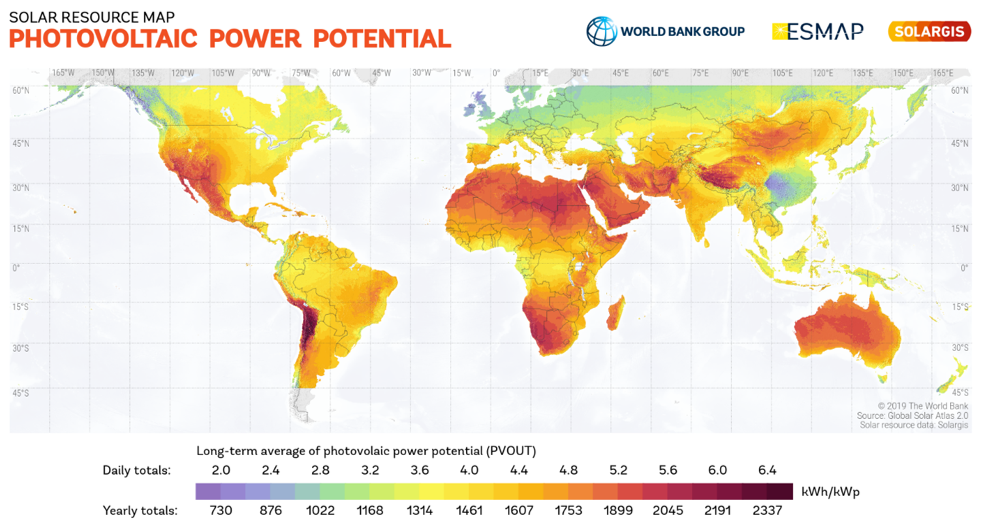


**Fig. S20** The map of photovoltaic power potential [S[1](#_ENREF_1_1)]

**Table S1** Energy Dispersive Spectroscopy (EDS) of Sn@Ti_2_SnC MAX

| **Element** | **Atomic %** |
| --- | --- |
| Ti | 19.02 |
| Sn | 10.03 |
| C | 37.93 |
| O | 33.02 |
| Totals | 100 |

**Table S2** Energy and cost analysis results

| Scenario | Content | Out-of-lab | Ideal | Cheap PV |
| --- | --- | --- | --- | --- |
| Scenario assumptions | NH_3_ yield rate (µg h^-1^ mg^-1^) | 10.53 | 26.37 | 26.37 |
|  | PV capital cost ($/W dc) | 0.26 | 0.26 | 0.13 |
|  | PV operational cost ($/kW/year) | 20.00 | 20.00 | 20.00 |
| Annual production | NH_3_ production (kg/year) | 1000 | 1000 | 1000 |
| Energy efficiency | Faradic efficiency (%) | 10.62 | 25.10 | 25.10 |
| PV-EC module | Number of PV module | 139 | 59 | 59 |
|  | Number of EC module | 4830 | 2040 | 2040 |
| Costs | Capital cost: PV module ($) | 46146 | 19523 | 9762 |
|  | Operational cost: PV module ($/year) | 1183 | 501 | 250 |
|  | Capital cost: EC module ($) | 13040 | 5508 | 5508 |
|  | Operational cost: EC module ($/year) | 435 | 184 | 184 |
|  | Total annualized cost ($/year) | 7181 | 3037 | 1869 |
| MSP | Minimum selling price ($/kg NH_3_) | 7.18 | 3.04 | 1.87 |

**Table S3** Design of one electrochemical (EC) module

| **Item** | **Material** |
| --- | --- |
| Cathode | 1 m^2^ carbon paper + 1.0 g catalyst |
| Anode | 1 m^2^ carbon paper |
| Electrolyte | 10 L 0.1 M Na_2_SO_4_ |
| Power (W) | 12.25 W: 2.5 V, 4.9 A |

**Table S4** Design of one residential photovoltaic (PV) module

| **Item** | **Description** |
| --- | --- |
| Module | SR-54M425NHL Pro (Mono-c-Si) |
| Nameplate DC capacity | 0.425 kW dc |
| Module area | 2.0 m^2^ (108 cells) |
| Solar resource | Tibet, China |
| Annual energy | 800 kWh dc (21.34% capacity factor) |

**Table S5** Major assumptions for economic analysis

| NH_3_ production rate | | 1000 kg/year |
| --- | --- | --- |
| Project lifetime | | 25 years |
| Discount rate | | 8% |
| **Purchase cost** | | |
| PV panel | | $0.26/W dc |
| PV-EC module | Catalyst | $200/kg |
|  | Carbon paper | $1/kg |
| Total capital cost* | | 300% Purchase cost |
| **Operation and maintenance cost** | | |
| PV panel | | $20/kW/year |
| PV-EC module | | 10% Purchase cost |

*Total capital cost includes costs of purchase, installation materials (30% of purchase cost), installation labor (48% of purchase cost), insurance and tax (10% of purchase cost), construction overhead (34% of purchase cost), engineering (7% of purchase cost), contingency and fee (41% of purchase cost) and site development (30% of purchase cost). The installation and engineering costs of the photovoltaic electrochemical (PV-EC) system are assumed lower than large-scale chemical plants due to the modularsystem design.

**Table S6** Energy and cost analysis results of out-of-lab conditions

| **Step 1: Design a single PV and EV module** | |
| --- | --- |
| PV module | |
| Module Name | SR-54M425NHLPro (Mono-c-Si) |
| Nameplate DC capacity (kW dc) | 0.425 |
| Module area | 2.0 m^2^ (108 cells) |
| Installation location | Tibet, China |
| Annual energy (kWh dc) | 800 |
| Capacity factor | 21.34% |
| Electrochemical (EC) module | |
| Cathode | 1 m^2^ carbon paper + 1.0 g catalyst |
| Anode | 1 m^2^ carbon paper |
| Electrolyte | 10 L 0.1 M Na_2_SO_4_ |
| Current (A) | 4.9 |
| Voltage (V) | 2.5 |
| NH_3_ yield (g/h) | 0.11 |
| Power (W) | 12.25 |
| NH_3_ energy utilization (g/kWh) | 8.98 |
| NH_3_ Faradic efficiency | 10.62% |
| **Step 2: Design an integrated PV-EC system** | |
| PV-EC system design | |
| NH_3_ production rate (kg/yr) | 1000 |
| Required system electricity (kWh dc) | 111364 |
| PV module # | 139 |
| Total PV Power (kW dc) | 59 |
| EC module # | 4830 |
| Constant | Value |
| Faradic's canstant (C/mol) | 96485 |
| Molecular weight NH_3_ (g/mol) | 17 |
| Electron transfer for NH_3_ | 3 |
| **Step 3: Cost analysis and NH_3_ MSP** | |
| Cost assumption | |
| PV capital cost ($/PV module) | 331.5 |
| PV operational cost ($/PV module/yr) | 8.5 |
| EC capital cost ($/PV module) | 2.7 |
| EC operational cost ($/PV module/yr) | 0.09 |
| Project lifetime (yr) | 25 |
| Annual discount rate | 8% |
| Annual capital recovery factor | 9.40% |
| Cost analysis results | |
| PV capital ($) | 46146 |
| PV operational ($/yr) | 1183 |
| EC capital ($) | 13040 |
| EC operational ($/yr) | 435 |
| Total annualized cost ($/yr) | 7181 |
| NH_3_ minimum selling price (MSP) ($/kg) | 7.18 |

**Table S7** Energy and cost analysis results of ideal scenario

| **Step 1: Design a single PV and EV module** | |
| --- | --- |
| PV module | |
| Module Name | SR-54M425NHLPro (Mono-c-Si) |
| Nameplate DC capacity (kW dc) | 0.425 |
| Module area | 2.0 m^2^ (108 cells) |
| Installation location | Tibet, China |
| Annual energy (kWh dc) | 800 |
| Capacity factor | 21.34% |
| Electrochemical (EC) module | |
| Cathode | 1 m^2^ carbon paper + 1.0 g catalyst |
| Anode | 1 m^2^ carbon paper |
| Electrolyte | 10 L 0.1 M Na_2_SO_4_ |
| Current (A) | 4.9 |
| Voltage (V) | 2.5 |
| NH_3_ yield (g/h) | 0.26 |
| Power (W) | 12.25 |
| NH_3_ energy utilization (g/kWh) | 21.22 |
| NH_3_ Faradic efficiency | 25.10% |
| **Step 2: Design an integrated PV-EC system** | |
| PV-EC system design | |
| NH_3_ production rate (kg/yr) | 1000 |
| Required system electricity (kWh dc) | 47115 |
| PV module # | 59 |
| Total PV Power (kW dc) | 25 |
| EC module # | 2040 |
| Constant | Value |
| Faradic's canstant (C/mol) | 96485 |
| Molecular weight NH_3_ (g/mol) | 17 |
| Electron transfer for NH_3_ | 3 |
| **Step 3: Cost analysis and NH_3_ MSP** | |
| Cost assumption | |
| PV capital cost ($/PV module) | 331.5 |
| PV operational cost ($/PV module/yr) | 8.5 |
| EC capital cost ($/PV module) | 2.7 |
| EC operational cost ($/PV module/yr) | 0.09 |
| Project lifetime (yr) | 25 |
| Annual discount rate | 8% |
| Annual capital recovery factor | 9.40% |
| Cost analysis results | |
| PV capital ($) | 19523 |
| PV operational ($/yr) | 501 |
| EC capital ($) | 5508 |
| EC operational ($/yr) | 184 |
| Total annualized cost ($/yr) | 3037 |
| NH_3_ minimum selling price (MSP) ($/kg) | 3.04 |

**Table S8** Energy and cost analysis results of cheap PV scenario

| **Step 1: Design a single PV and EV module** | |
| --- | --- |
| PV module | |
| Module Name. | SR-54M425NHLPro (Mono-c-Si) |
| Nameplate DC capacity (kW dc) | 0.425 |
| Module area | 2.0 m^2^ (108 cells) |
| Installation location | Tibet, China |
| Annual energy (kWh dc) | 800 |
| Capacity factor | 21.34% |
| Electrochemical (EC) module | |
| Cathode | 1 m^2^ carbon paper + 1.0 g catalyst |
| Anode | 1 m^2^ carbon paper |
| Electrolyte | 10 L 0.1 M Na_2_SO_4_ |
| Current (A) | 4.9 |
| Voltage (V) | 2.5 |
| NH_3_ yield (g/h) | 0.26 |
| Power (W) | 12.25 |
| NH_3_ energy utilization (g/kWh) | 21.22 |
| NH_3_ Faradic efficiency | 25.10% |
| **Step 2: Design an integrated PV-EC system** | |
| PV-EC system design | |
| NH_3_ production rate (kg/yr) | 1000 |
| Required system electricity (kWh dc) | 47115 |
| PV module # | 59 |
| Total PV Power (kW dc) | 25 |
| EC module # | 2040 |
| Constant | Value |
| Faradic's canstant (C/mol) | 96485 |
| Molecular weight NH_3_ (g/mol) | 17 |
| Electron transfer for NH_3_ | 3 |
| **Step 3: Cost analysis and NH_3_ MSP** | |
| Cost assumption | |
| PV capital cost ($/PV module) | 165.75 |
| PV operational cost ($/PV module/yr) | 4.25 |
| EC capital cost ($/PV module) | 2.7 |
| EC operational cost ($/PV module/yr) | 0.09 |
| Project lifetime (yr) | 25 |
| Annual discount rate | 8% |
| Annual capital recovery factor | 9.40% |
| Cost analysis results | |
| PV capital ($) | 9762 |
| PV operational ($/yr) | 250 |
| EC capital ($) | 5508 |
| EC operational ($/yr) | 184 |
| Total annualized cost ($/yr) | 1869 |
| NH_3_ minimum selling price (MSP) ($/kg) | 1.87 |

**Table S9** Comparison of electrocatalytic N_2_ reduction performance among reported Sn-based and MXene-based NRR catalysts under ambient conditions

| **Catalyst** | **System** | **Yield** | **FE** | **Reference** |
| --- | --- | --- | --- | --- |
| Sn@Ti_2_CT_X_/Ti_2_SnC-V_Sn_ | 0.1 M Na_2_SO_4_ | 28.4 μg h^-1^ mg^-1^ | 15.57% | This work |
| Sn_sc_/C | 0.1 M Na_2_SO_4_ | 17.28 μg h^–1^ mg^-1^ | 22.76% | [S[2](#_ENREF_1_2)] |
| Sn/SnS_2_ | 0.1 M NaOH | 23.8 μg h^-1^ mg^-1^ | 3.4% | [S[3](#_ENREF_1_3)] |
| Sn dendrites | 0.1 M PBS | 5.66 × 10^-11^ mol s^-1^ cm^-2^ | 3.67% | [S[4](#_ENREF_1_4)] |
| Sn@MoSe_2_@C NFMs | 0.1 M Na_2_SO_4_ | 1.61 × 10^−10^ mol s^-1^ cm^-2^ | 14.51% | [S[5](#_ENREF_1_5)] |
| Sn-BPene | 0.1 M PBS | 26.98 µg h^-1^ mg_cat_^-1^ | 36.51% | [S[6](#_ENREF_1_6)] |
| F-SnO_2_/CC | 0.1 M Na_2_SO_4_ | 19.3 µg h^-1^ mg^-1^ | 8.60% | [S[7](#_ENREF_1_7)] |
| Fe-doped SnO_2_ | 0.1 M Na_2_SO_4_ | 28.45 µg h^-1^ mg_cat_^-1^ | 6.54% | [S[8](#_ENREF_1_8)] |
| SnO_2_-O_v_ | 0.1 M Na_2_SO_4_ | 25.27 µg h^-1^ mg_cat_^-1^ | 11.48 % | [S[9](#_ENREF_1_9)] |
| Sn ADP-0.38 | 0.1 M Na_2_SO_4_ | 28.3 µg h^-1^ mg^-1^ | 26.8% | [S[10](#_ENREF_1_10)] |
| Ti_3_C_2_T_X_ (T=F, OH) | 0.1 M HCl | 20.4 µg h^-1^ mg_cat_^-1^ | 9.30% | [S[11](#_ENREF_1_11)] |
| Ti_3_C_2_OH QDs | 0.1 M HCl | 62.94 μg h^-1^ mg^-1^ | 13.30% | [S[12](#_ENREF_1_12)] |
| V_2_CT_X_ | 0.1 M Na_2_SO_4_ | 12.6 µg h^-1^ mg_cat_^-1^ | 4% | [S[13](#_ENREF_1_13)] |
| Mo_2_CT_X_ | 0.5 M K_2_SO_4_ | 10.43 µg h^-1^ mg_cat_^-1^ | 7.73% | [S[14](#_ENREF_1_14)] |
| Mo_2_C/C | 0.5 M Li_2_SO_4_ | 11.3 µg h^-1^ mg_cat_^-1^ | 7.80% | [S[15](#_ENREF_1_15)] |
| V_4_C_3_T_X_ | 0.1 M KOH | 20.41 µg h^-1^ mg_cat_^-1^ | 10.11% | [S[16](#_ENREF_1_16)] |
| Ni@V_4_C_3_T_X_ | 0.1 M KOH | 21.29 µg h^-1^ mg_cat_^-1^ | 14.86% | [S[16](#_ENREF_1_16)] |

**Supplementary References**

S1. Solargis. The World Bank, Solaresource data, https://www.globalsolaratlas.info, (2019).

S2. Z. Xue, C. Sun, M. Zhao, Y. Cui, Y. Qu, H. Ma, Z. Wang, Q. Jiang. Efficient electrocatalytic nitrogen reduction to ammonia on ultrafine Sn nanoparticles. ACS Appl Mater Interfaces. **13**(50), 59834–59842 (2021). <https://doi.org/10.1021/acsami.1c15324>

S3. P. Li, W. Fu, P. Zhuang, Y. Cao, C. Tang, A. B. Watson, P. Dong, J. Shen, M. Ye. Amorphous Sn/crystalline SnS_2_ nanosheets via in situ electrochemical reduction methodology for highly efficient ambient N_2_ fixation. Small. **15**(40), 1902535 (2019). <https://doi.org/10.1002/smll.201902535>

S4. X. Lv, F. Wang, J. Du, Q. Liu, Y. Luo, S. Lu, G. Chen, S. Gao, B. Zheng, X. Sun. Sn dendrites for electrocatalytic N_2_ reduction to NH_3_ under ambient conditions. Sustainable Energy Fuels. **4**(9), 4469-4472 (2020). <https://doi.org/10.1039/d0se00828a>

S5. S. Huang, M. Zhang, Y.-T. Liu. Preparation and NRR application of transition metal nanosheets on carbon nanofiber membranes. J Phys: Conf Ser. **1948**, (2021). <https://doi.org/10.1088/1742-6596/1948/1/012222>

S6. H. Liu, X. Cao, L. X. Ding, H. Wang. Sn-doped black phosphorene for enhancing the selectivity of nitrogen electroreduction to ammonia. Adv Funct Mater. **32**(19), (2022). <https://doi.org/10.1002/adfm.202111161>

S7. Y.-p. Liu, Y.-b. Li, H. Zhang, K. Chu. Boosted electrocatalytic N_2_ reduction on fluorine-doped SnO_2_ mesoporous nanosheets. Inorg Chem. **58**(15), 10424–10431 (2019). <https://doi.org/10.1021/acs.inorgchem.9b01823>

S8. Y. Li, Y. Liu, X. Liu, Y. Liu, Y. Cheng, P. Zhang, P. Deng, J. Deng, Z. Kang, H. Li. Fe-doped SnO_2_ nanosheet for ambient electrocatalytic nitrogen reduction reaction. Nano Res. **15**, 6026-6035 (2022). <https://doi.org/10.1007/s12274-022-4298-2>

S9. X. He, H. Guo, T. Liao, Y. Pu, L. Lai, Z. Wang, H. Tang. Electrochemically synthesized SnO_2_ with tunable oxygen vacancies for efficient electrocatalytic nitrogen fixation. Nanoscale. **13**(38), 16307-16315 (2021). <https://doi.org/10.1039/d1nr04621g>

S10. L. Zhang, H. Zhou, X. Yang, S. Zhang, H. Zhang, X. Yang, X. Su, J. Zhang, Z. Lin. Boosting electroreduction kinetics of nitrogen to ammonia via atomically dispersed Sn protuberance. Angew Chem, Int Ed. **62**(13), e202217473 (2023). <https://doi.org/10.1002/anie.202217473>

S11. J. Zhao, L. Zhang, X.-Y. Xie, X. Li, Y. Ma, Q. Liu, W.-H. Fang, X. Shi, G. Cui, X. Sun. Ti_3_C_2_T_x_ (T = F, OH) MXene nanosheets: conductive 2D catalysts for ambient electrohydrogenation of N_2_ to NH_3_. J Mater Chem A. **6**(47), 24031-24035 (2018). <https://doi.org/10.1039/c8ta09840a>

S12. Z. Jin, C. Liu, Z. Liu, J. Han, Y. Fang, Y. Han, Y. Niu, Y. Wu, C. Sun, Y. Xu. Rational design of hydroxyl‐rich Ti_3_C_2_T_x_ MXene quantum qots for high‐performance electrochemical N_2_ reduction. Adv Energy Mater. **10**(22), (2020). <https://doi.org/10.1002/aenm.202000797>

S13. J. Xia, H. Guo, G. Yu, Q. Chen, Y. Liu, Q. Liu, Y. Luo, T. Li, E. Traversa. 2D vanadium carbide (MXene) for electrochemical synthesis of ammonia under ambient conditions. Catal Lett. **151**, 3516–3522 (2021). <https://doi.org/10.1007/s10562-021-03589-6>

S14. W. Peng, M. Luo, X. Xu, K. Jiang, M. Peng, D. Chen, T. S. Chan, Y. Tan. Spontaneous atomic ruthenium doping in Mo_2_CT_X_ MXene defects enhances electrocatalytic activity for the nitrogen reduction reaction. Adv Energy Mater. **10**(25), 2001364 (2020). <https://doi.org/10.1002/aenm.202001364>

S15. H. Cheng, L.-X. Ding, G.-F. Chen, L. Zhang, J. Xue, H. Wang. Molybdenum carbide nanodots enable efficient electrocatalytic nitrogen fixation under ambient conditions. Adv Mater. **30**(46), 1803694 (2018). <https://doi.org/10.1002/adma.201803694>

S16. C.-F. Du, L. Yang, K. Tang, W. Fang, X. Zhao, Q. Liang, X. Liu, H. Yu, W. Qi, Q. Yan. Ni nanoparticles/V_4_C_3_T_x_ MXene heterostructures for electrocatalytic nitrogen fixation. Mater Chem Front. **5**(5), 2338-2346 (2021). <https://doi.org/10.1039/d0qm00898b>
